# Pharmacological reversion of sphingomyelin-induced dendritic spine anomalies in a Niemann Pick disease type A mouse model

**DOI:** 10.1002/emmm.201302649

**Published:** 2014-01-21

**Authors:** Ana I Arroyo, Paola G Camoletto, Laura Morando, Marco Sassoe-Pognetto, Maurizio Giustetto, Paul P Van Veldhoven, Edward H Schuchman, Maria D Ledesma

**Affiliations:** 1 Department of Neurobiology, Centro Biologia Molecular Severo Ochoa, CSIC-UAMMadrid, Spain; 2 Department of Neuroscience, National Institute of Neuroscience-Italy, University of TurinTurin, Italy; 3 Department of Cellular and Molecular Medicine, LIPIT, Katholieke Universiteit LeuvenLeuven, Belgium; 4 Department of Genetics and Genomic Sciences, Mount Sinai School of Medicine, Icahn Medical InstituteNew York, NY, USA

**Keywords:** dexamethasone, Niemann Pick, RhoA, sphingomyelin

## Abstract

Understanding the role of lipids in synapses and the aberrant molecular mechanisms causing the cognitive deficits that characterize most lipidosis is necessary to develop therapies for these diseases. Here we describe sphingomyelin (SM) as a key modulator of the dendritic spine actin cytoskeleton. We show that increased SM levels in neurons of acid sphingomyelinase knock out mice (ASMko), which mimic Niemann Pick disease type A (NPA), result in reduced spine number and size and low levels of filamentous actin. Mechanistically, SM accumulation decreases the levels of metabotropic glutamate receptors type I (mGluR1/5) at the synaptic membrane impairing membrane attachment and activity of RhoA and its effectors ROCK and ProfilinIIa. Pharmacological enhancement of the neutral sphingomyelinase rescues the aberrant molecular and morphological phenotypes *in vitro* and *in vivo* and improves motor and memory deficits in ASMko mice. Altogether, these data demonstrate the influence of SM and its catabolic enzymes in dendritic spine physiology and contribute to our understanding of the cognitive deficits of NPA patients, opening new perspectives for therapeutic interventions.

**Subject Categories** Genetics, Gene Therapy & Genetic Disease; Neuroscience

## Introduction

Alterations in dendritic spines, protrusions at the postsynaptic membrane that receive most of the excitatory input in the central nervous system (Yuste ' Tank, 1996), have been related to many cognitive disorders (Carlisle ' Kennedy, 2005). Dynamic changes in spine shape, size and number upon stimuli is essential in learning and memory processes (Yuste ' Bonhoeffer, 2001). The actin cytoskeleton, enriched in the spines, regulates the spine dynamism (Frost *et al*, 2010). Intense research in recent years has led to a detailed knowledge on the protein machinery interacting with actin that modulates the dynamics of spine morphology, which includes extracellular ligands, neurotransmitter receptors, scaffold proteins, the Rho family of small GTPases and proteins that directly control actin polymerization (Tada ' Sheng, 2006). However, much less is known about the role of lipids in these processes. This is especially relevant considering that the remodelling of the postsynaptic membrane, of which lipids are major components, is as remarkable as that of the underlying cytoskeleton in spine plasticity. Moreover, the activity of key proteins in synaptic remodelling depends on their interaction with the membrane. Further support for a key role of lipids in spine dynamics comes from the fact that genetic defects affecting lipid metabolism, and leading to lipidosis, frequently cause cognitive impairment (Futermann ' Van Meer, 2004).

Sphingolipids are major components of neuronal membranes, where they are particularly enriched (Schwarz *et al*, 1995). Mounting evidence indicates that these lipids actively participate in essential functions including signaling (Simons ' Toomre, 2000), proteolysis (Ledesma *et al*, 2003), endocytosis (Parton ' Richards, 2003) and the establishment and maintenance of axonal polarity (Ledesma *et al*, 1999; Galvan *et al*, 2005). Sphingolipids are also involved in the formation and/or maintenance of dendritic spines. Thus, pharmacological inhibition of sphingolipids led to dendritic spine alterations in cultured primary hippocampal neurons (Hering *et al*, 2003). In addition, biochemical and microscopy studies have indicated that the localization of several postsynaptic proteins, including scaffold proteins and neurotransmitter receptors, also depend on sphingolipids (Bruses *et al*, 2001; Hering *et al*, 2003). However, the molecular mechanisms by which sphingolipids exert these effects are largely unknown.

Niemann Pick disease type A (NPA) is a sphingolipidosis caused by loss of function mutations in the *SMPD1* gene encoding for the acid sphingomyelinase (ASM). NPA leads to severe and early onset neurodegeneration (Brady *et al*, 1966) for which no treatment is yet available. ASM participates in sphingolipid metabolism by hydrolyzing SM (Stoffel, 1999). In mice that lack this enzyme (ASMko), whose phenotype mimics the human disease (Horinouchi *et al*, 1995), SM accumulates at the neuronal plasma membrane leading to impaired endocytosis and mislocalization of GPI-anchored proteins (Galvan *et al*, 2008). Moreover, accumulation of SM and its derivative sphingosine also occurs at the presynaptic membranes of mutant mice causing alterations in synaptic vesicle docking and presynaptic plasticity events (Camoletto *et al*, 2009). In the present study we have analyzed ASMko mice to investigate whether and how altered sphingolipid levels affect dendritic spines and if sphingolipid modulation could become a suitable treatment for NPA. The results indicate that high SM levels due to ASM deficiency reduce the number and size of dendritic spines. In addition, we provide evidence for the molecular mechanism underlying these alterations and novel strategies to revert them *in vitro* and *in vivo*.

## Results

### Lack of ASM reduces dendritic spine number and size

**Figure 1:**
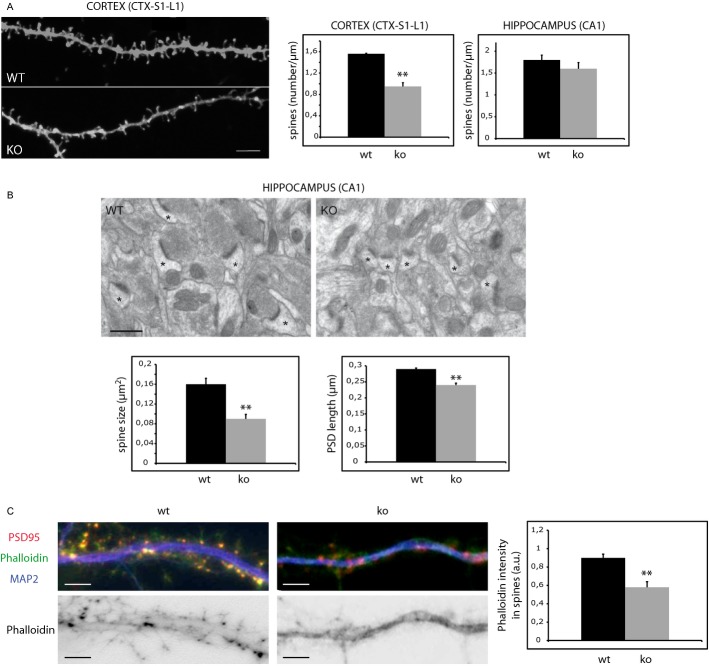
Aberrant dendritic spines and low levels of filamentous actin in ASMko neurons.
Dendritic spines in dendrites of neurons from the S1-L1 cortex of wt and ASMko mice visualized by diOlistics and confocal microscopy. Graphs show dendritic spine density per μm of dendritic segments in the S1-L1 cortex (*P* = 0.01) or the CA1 region of the hippocampus (*n* = 4). Bar 5 μm.Electron micrographs of synapses in the hippocampal CA1 stratum radiatum of wt and ASMko mice. Spines are indicated by asterisks. Graphs show mean and standard deviation (mean ± s.d.) of spine size in μm^2^ (*P* = 0.016) and PSD length in μm (*P* = 0.006) in wt and ASMko mice (*n* = 70 synapses in each of 3 mice per genotype).Top: Dendrites from wt or ASMko cultured hippocampal neurons stained for MAP2 (blue), PSD95 (red), and phalloidin (green); bottom: phalloidin staining only. The graph shows mean ± s.d. of phalloidin fluorescence intensity per spine area (*n* = 250 dendritic spines from 3 independent cultures, *P* = 0,011). Bars: 5 μm.

To investigate the effects on dendritic spines of loss of function mutations of the *SMPD1* gene, encoding for ASM, we analyzed these structures *in vivo* by diOlistic fluorescent labelling of brain sections through the S1 cortex and the hippocampal formation of age-matched wild type (wt) and ASMko mice. We chose to analyze these brain areas because of their involvement in learning and memory abilities, which are impaired in NPA patients. Confocal stacks z-projections from segments of secondary apical dendrites of somatosensory cortical and CA1 hippocampal pyramidal neurons were used for the quantitative analyses of dendritic spines (Fig 1A). The number of spines identified with DiI labelling per micrometer of dendrite length was significantly reduced in the layer 1 (L1) of the S1 cortex of ASMko brains compared to wt (wt: 1.56 ± 0.01 spines/μm; ASMko: 0.95 ± 0.07 spines/μm). Although there was a similar tendency to reduction in the CA1 pyramidal neurons of the hippocampus in ASMko mice the difference in the number of dendritic spines between genotypes was not statistically significant in this area (wt: 1.80 ± 0.11 spines/μm; ASMko: 1.60 ± 0.14 spines/μm). To accurately analyze not only the number but also the size of the spines, we performed electron microscopy analysis in *stratum radiatum* of the hippocampal CA1 region of age matched wt and ASMko mice (Fig 1B). While the number of spines did not change significantly (wt: 2.20 ± 0.3 synapses/μm^3^; ASMko: 2.22 ± 0.29 synapses/μm^3^), in agreement with the diOlistic analysis data, the area of the postsynaptic compartment was smaller in ASMko conditions (wt: 0.16 ± 0.012 μm^2^; ASMko: 0.09 ± 0.009 μm^2^). Accordingly, the length of the postsynaptic density was significantly reduced (wt: 0.29 ± 0.003 μm; ASMko: 0.24 ± 0.006 μm). Dendritic spine size was also reduced in the cerebellum of ASMko mice (wt: 0.33 ± 0.002 μm; ASMko: 0.27 ± 0.002 μm) (Supplementary Fig 1A). Altogether these results show that the absence of ASM has a broad impact in dendritic spines of different neuronal populations ranging from spine loss in the cortex to reduced size in the hippocampus and cerebellum.

### Reduced levels of filamentous actin in ASMko dendritic spines due to SM accumulation

**Figure 2:**
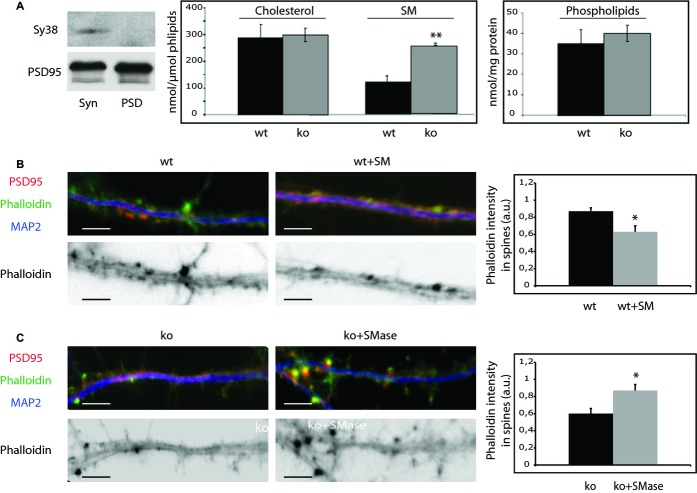
High SM levels accumulate in ASMko postsynaptic membranes and reduce the amount of filamentous actin. A Western blots of the presynaptic and postsynaptic markers synaptophysin (Sy38) and PSD 95, respectively, in extracts containing the same amount of protein from total synaptosomal preparation (Syn) and from the postsynaptic enriched fraction (PSD). Graphs show mean ± s.d. of the levels of SM (*P* = 0.011) and cholesterol (in nmol/μmol phospholipids) and of phospholipids (nmol/mg protein) in postsynaptic membranes (PSD fraction) of wt and ASMko mice (*n* = 6). B,C Top: Dendrites from cultured hippocampal neurons from wt mice treated or not with SM (B) or from ASMko mice treated or not with SMase (C) stained for MAP2 (blue), PSD95 (red), and phalloidin (green); bottom: phalloidin staining only. The graphs show mean ± s.d. of phalloidin fluorescence intensity per spine area (*n* = 250 dendritic spines from 3 independent cultures, *P_wt+__SM_ = 0.02; *P_ko+Smase_ = 0.03). Bars: 5 μm.

Because of the key role of the actin cytoskeleton in dendritic spine size we compared actin polymerization in primary hippocampal neuron cultures derived from wt and ASMko mice by phalloidin staining. Phalloidin associated fluorescence, which is specific for filamentous actin, was 1.55-fold reduced in ASMko compared to wt dendritic spines (Fig 1C). Our next aim was to understand the molecular mechanism underlying this aberrant phenotype. Lack of ASM leads to the accumulation of SM in total synaptosomal membranes (Camoletto *et al*, 2009). To test to which extent this affects the postsynaptic compartment an additional step was taken in the synaptosome isolation protocol so to obtain a fraction highly enriched in postsynaptic membranes (Schubert *et al*, 2006) (Fig 2A). Mass analysis of different lipids in this fraction (see methods) indicated that while the levels of phospholipids (wt: 35 ± 7 nmol/mg protein; ASMko: 40 ± 4 nmol/mg protein) or cholesterol (wt: 287 ± 52 nmol/μmol phospholipids; ASMko: 298 ± 25 nmol/μmol phospholipids) did not show significant differences, a two-fold increase in SM (wt: 108 ± 21 nmol/μmol phospholipids; ASMko: 225 ± 4 nmol/μmol phospholipids) occurred in the postsynaptic fraction obtained from ASMko brains compared to wt (Fig 2A). To test whether high SM levels could account for the actin alterations observed in the spines, this lipid was added to cultured hippocampal neurons from wt mice. We increased SM amount without significantly affecting levels of cholesterol/phospholipids or the viability of the cells (see Materials and Methods and Galvan *et al*, 2008). Phalloidin staining in the spines was 1.38-fold diminished compared to non-treated neurons (Fig 2B). In addition, ASMko cultured neurons were treated with exogenous sphingomyelinase. This treatment restored SM amount to levels similar to wt without changing cholesterol/phospholipid amount or inducing cell death (see Materials and Methods and Galvan *et al*, 2008). Phalloidin staining in spines was 1.45-fold increased reaching levels similar to those in wt neurons (Fig 2C). These findings indicated a direct relationship between SM accumulation and dendritic spine alterations. Moreover, they pointed to low levels of filamentous actin as a cause for the reduced size of ASMko dendritic spines.

### Membrane attachment of RhoA and its effectors are reduced in ASMko synaptosomes due to high SM levels

**Figure 3:**
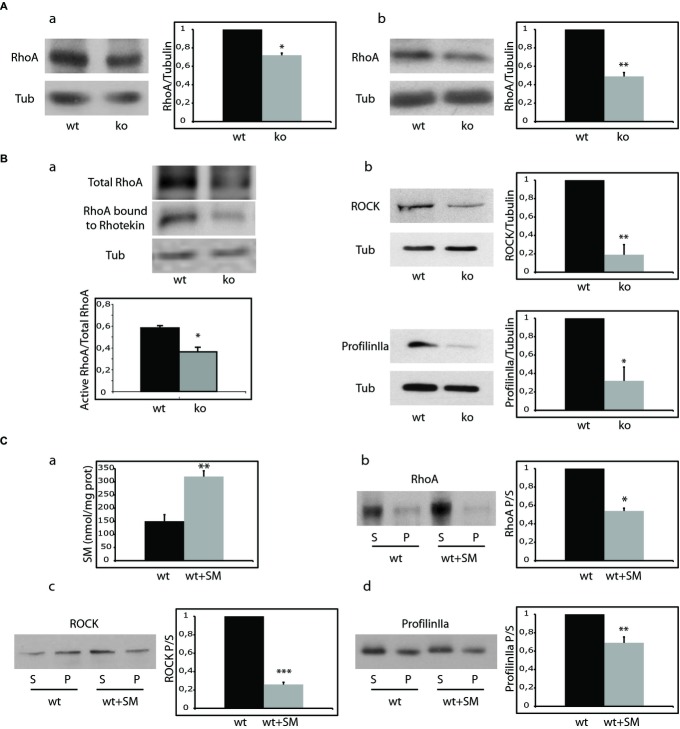
Absence of ASM and SM modulation alter the levels and activity of RhoA and its effectors in synaptosomes.
Western blot of RhoA and tubulin levels in total (a) and membrane extracts (b) from wt and ASMko synaptosomes. Graphs show mean ± s.d. of RhoA levels in ASMko conditions normalized to tubulin and referred to wt levels that were considered as 1 (*n* = 3, *P_total RhoA_ = 0.04, *P_membrane RhoA_ = 0.008).(a) Activity of RhoA in wt and ASMko synaptosomes determined by the Rhotekin binding assay. Tubulin is shown as loading control. Graph shows mean ± s.d. of the ratio of Rhotekin-bound (active) RhoA to total RhoA (*n* = 3, *P = 0.025). (b) Western blots of ROCK, ProfilinIIa and tubulin levels in membrane extracts from wt and ASMko synaptosomes. Graphs show mean ± s.d. of ROCK (*P = 0.017) or ProfilinIIa (*P = 0.033) levels in ASMko conditions normalized to tubulin and referred to wt levels that were considered as 1 (*n* = 3).(a) SM levels (nmol/mg protein) in wt synaptosomes treated or not with SM. Graph shows mean ± s.d. in treated synaptosomes referred to non treated that were considered as 1 (*n* = 3, **P = 0.019). (b, c, d) Western blots of RhoA (b), ROCK (c) and ProfilinIIa (d) levels in supernatants (S) and pellets (*P*) after 100,000 *g* centrifugation of wt synaptosomes treated or not with SM. Graphs show mean ± s.d. of each protein ratio pellet/supernatant in treated samples referred to non-treated that were considered as 1 (*n* = 3; *P_R__hoASM_ = 0.029, ***P_ROCKSM_ = 0.0009, **P_profilinIIaSM_= 0.008).

The small GTPases of the Rho family, cdc42, RhoA and Rac1 and their effectors are major modulators of actin polymerization in dendritic spines (Tada ' Sheng, 2006). To test whether alterations in these proteins could account for the low levels of filamentous actin found in ASMko spines, their amount and membrane attachment, which is necessary for their activation (Buchsbaum, 2007), were compared in synaptosomal fractions of wt and ASMko mice brains. Total levels and membrane attachment were similar for cdc42 and Rac1 (Supplementary Fig 2). In contrast, total RhoA levels in ASMko conditions were reduced (28% lower than in wt) (Fig 3Aa). A greater reduction (51%) was evident in the amount of RhoA bound to the membrane (Fig 3Ab). Consistently, we found low amounts of active RhoA, determined by the ability to bind Rhotekin (38% less RhoA bound to Rhotekin in ASMko samples compared to wt) (Fig 3Ba). Because RhoA enhances the stability of filamentous actin in dendritic spines through complexing with its downstream effectors RhoA-specific kinase (ROCK) and profilinIIa (Schubert *et al*, 2006) we monitored the membrane-bound amount of these molecules. In agreement with reduced filamentous actin, ASMko synaptosomal membranes presented significantly lower levels of ROCK and profilinIIa than those wt (81 and 68% reductions, respectively) (Fig 3Bb). To investigate if the alterations in the RhoA pathway could be due to SM accumulation we modulated the levels of this lipid. On one hand, we added SM to wt synaptosomes achieving a 2.1- fold increase in the lipid levels of synaptic membranes similar to the ASMko situation (Fig 3Ca) (see methods and Camoletto *et al*, 2009). SM addition resulted in 46% reduction of RhoA membrane attachment (Fig 3Cb). The levels of membrane-bound RhoA effectors ROCK and profilinIIa were also reduced significantly (79 and 31%, respectively) (Fig 3Cc,d). On the other hand, sphingomyelinase treatment of ASMko synaptosomes reduced SM levels and increased RhoA membrane binding by 3- and 4.5- fold, respectively (Supplementary Fig 3).

Altogether these data show the influence in synapses of SM levels in the RhoA pathway, which is a key modulator of actin polimerization in dendritic spines (Schubert *et al*, 2006).

### mGluR1/5 levels and interaction with RhoA are reduced in ASMko synaptosomes

**Figure 4:**
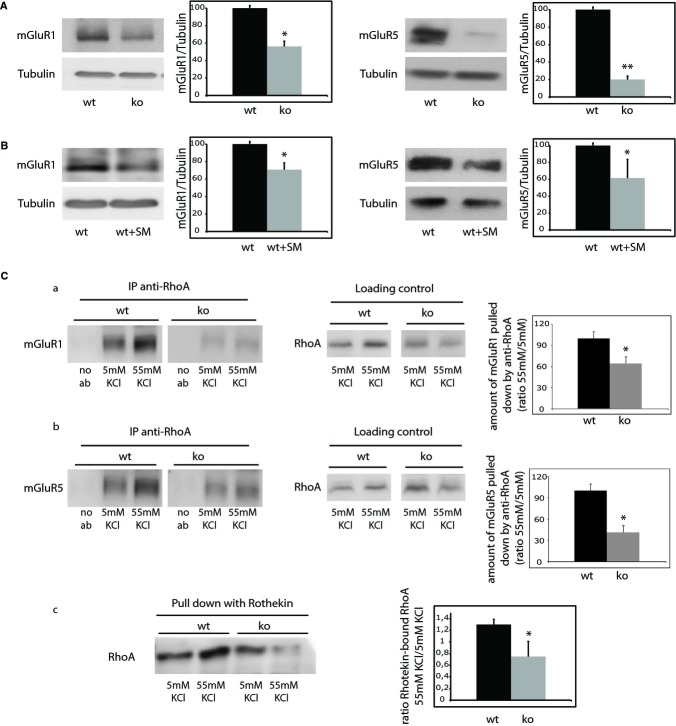
Levels of mGluR1 and mGluR5 and their interaction with RhoA upon stimuli are diminished in ASMko synaptosomes.
Western blot of mGluR1/5 and tubulin levels in membrane extracts of wt and ASMko synaptosomes. Graphs show mean ± s.d. in ASMko conditions normalized to tubulin and referred to wt levels that were considered as 100% (*n* = 3; *P_mGluR1_ = 0.031, **P_mGluR5_ = 0.02).Western blot of mGluR1/5 levels in wt synaptosomes treated or not with SM. Graphs show mean ± s.d. of mGluR1/5 levels normalized to tubulin in SM treated samples referred to those non treated that were considered as 100% (*n* = 3; *P_mGluR1_ = 0.034, *P_mGluR5_ = 0.041).Levels of interaction of mGluR1 (a) or mGluR5 (b) with RhoA as determined by immunoprecipitation of mGluR1/5 using the antibody against RhoA in wt and ASMko synaptosomes in control conditions (5 mM KCl) or upon stimulus (55 mM KCl). Specificity of the immunoprecipitation was monitored in extracts not incubated with anti-RhoA (no ab). Loading controls show the total amount of RhoA in the samples used for the immunoprecipitation assays. Graphs show mean ± s.d. in arbitrary units of the amount of mGluR1/5 pulled down by the anti-RhoA antibody (*n* = 3; *P_mGluR1_ = 0.04, *P_mGluR5_ = 0.023). (c) Changes in the activity of RhoA determined by the Rhotekin binding assay in synaptosomes from wt and ASMko mice brains stimulated (55 mM KCl) or not (5 mM KCl) with KCl. Graph shows mean ± s.d. of stimulus-induced RhoA activation as the ratio of Rhotekin-bound RhoA in 55/5 mM in wt or ASMko samples (*n* = 3, *P = 0.035).

RhoA can associate with the plasma membrane in dendritic spines through its interaction with Group I metabotropic glutamate receptors (mGluR1/5). This interaction is enhanced upon stimuli (Schubert *et al*, 2006). Moreover, mGluR1/5 localize to cholesterol-sphingolipid membrane domains (Francesconi *et al*, 2009), which show altered composition in ASMko neuronal membranes (Galvan *et al*, 2008). Hence, we hypothesized that alterations in mGluR1/5 could account for the reduced RhoA attachment to the ASMko synaptic membrane and thus activation. To test this hypothesis, the levels of these receptors were compared in wt and ASMko synaptosomes. Both mGluR1 and mGluR5 showed a significant reduction in ASMko conditions (43 and 80%, respectively) (Fig 4A). That increased SM levels are responsible for such deficiency was strongly supported by the 29 and 39% decrease found in the levels of mGluR1 and mGluR5, respectively, in wt synaptosomes treated with this lipid compared to non-treated synaptosomes (Fig 4B). To further assess our hypothesis on the altered interaction between mGluR1/5 and RhoA we performed immunoprecipitation assays. We observed that the enhanced RhoA-mGluR1/5 interaction in wt synaptosomes upon stimulation was not achieved in stimulated ASMko synaptosomes (Fig 4Ca,b). Consistently, RhoA activity increased upon stimulation in wt synaptosomes (1.3-fold respect to non-stimulated wt synaptosomes) as demonstrated by a Rhotekin binding assay. This stimulus-induced increase in active RhoA levels did not occur in ASMko conditions (0.75-fold in stimulated with respect to non-stimulated ASMko synaptosomes) (Fig 4Cc). Altogether, these results point to the contribution of SM-induced reduction of mGluR1/5 levels to the impaired RhoA membrane attachment and activation in ASMko synapses.

### Reduction of SM levels by activation of neutral sphingomyelinase (NSM) restores RhoA membrane binding and filamentous actin levels in ASMko synapses *in vitro*

**Figure 5:**
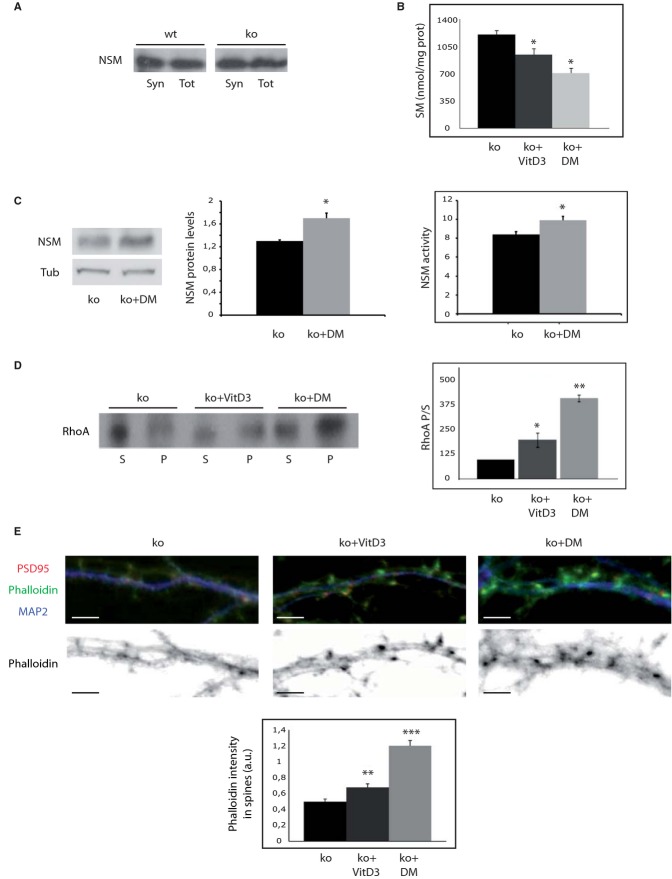
*In vitro* treatments with 1α, 25-dihydroxivitamin D3 or dexamethasone diminish SM amount, increase NSM protein levels and activity, and restore RhoA membrane binding and filamentous actin levels in ASMko synapses.
Western blot of NSM protein levels in total (Tot) and synaptosomal (Syn) fractions from wt and ASMko mice brains containing the same amount of protein.Mean ± s.d. of SM levels (nmol/mg protein) in ASMko synaptosomes treated or not with 1α, 25-dihydroxivitamin D3 (VitD3) or dexamethasone (DM) (*n* = 3, *P_vitD3_ = 0.04; *P_DM_=0.033).Western blot of NSM and tubulin levels in ASMko synaptosomes treated or not with VitD3 and dexamethasone. Graph shows mean ± s.d. of NSM protein levels normalized to tubulin (*n* = 3, *P* = 0.025). Graphs to the right show mean ± s.d. of NSM activity in ASMko synaptosomes treated or not with dexamethasone (*n* = 3, *P = 0.032).Western blots of RhoA levels in supernatants (S) and pellets (*P*) after 100,000 *g* centrifugation of ASMko synaptosomes treated or not with VitD3 or DM. Graph shows mean ± s.d. of the RhoA ratio pellet/supernatant in the treated samples as percentage of ASMko non treated samples that were considered 100% (*n* = 3; *P_vitD3_ = 0.042; **P_DM_ = 0.001).Top: Dendrites from ASMko neurons non treated or treated with vitaminD3 or dexamethasone stained for MAP2 (blue), PSD95 (red), and phalloidin (green); bottom: phalloidin staining only. The graph shows mean ± s.d. of phalloidin fluorescence intensity per spine area (*n* = 250 dendritic spines from 3 independent cultures, **P_vitD3_ = 0.001; ***P_DM_ = 0.0008). Bars: 5 μm.

The results reported so far pointed to SM accumulation at the synaptic membrane as responsible for the alterations in RhoA leading to cytoskeletal actin anomalies in dendritic spines lacking ASM. To further demonstrate this point and to search for rescue strategies, we next aimed to reduce SM levels by activating NSM, which is the main responsible for SM hydrolysis at the plasma membrane (Stoffel, 1999) and contributes to synaptic plasticity (Wheeler *et al*, 2009). To determine whether NSM could be a suitable target to modulate SM levels at ASMko synaptic membranes we first determined the levels of this enzyme at synapses. NSM showed similar levels at synaptic and total membranes from wt and ASMko mice brains (Fig 5A). The active form of Vitamin D3 (1α, 25-dihydroxyvitamin D3) and the synthetic steroid hormone dexamethasone have been shown to increase NSM activity, reducing SM levels in non neuronal cell cultures (Okazaki *et al*, 1989; Ramachandran *et al*, 1990). Hence, we incubated ASMko synaptosomes with 0.1 μM 1α, 25-dihydroxyvitamin D3 or dexamethasone for 1 h at 37°C. The treatments resulted in 25 and 41% decrease in SM levels, respectively (Fig 5B). Indicative of the involvement of NSM in these effects we observed a significant 30 and 15% increase in NSM protein and activity levels, respectively, upon dexamethasone treatment (Fig 5C) (NSM protein levels were also increased (17%) by 1α, 25-dihydroxyvitamin D3 treatment although in this case the change was not significant). 1α, 25-dihydroxyvitamin D3 and dexamethasone treatments enhanced RhoA binding to the ASMko synaptic membrane by 1.98 and 4-fold, respectively (Fig 5D) but had no effect in synaptosomes derived from wt mice where SM levels were also unaltered (Supplementary Fig 4A and B).

To assess the effect of enhanced NSM levels and SM reduction on actin polymerization we incubated cultured hippocampal neurons derived from ASMko mice with 0.1 μM 1α, 25-dihydroxyvitamin D3 or dexamethasone. The treatments started at 9 days *in vitro* (DIV) and went on until 15DIV when cultured neurons are fully mature and dendritic spines are evident. We observed 39 and 117% increments in the filamentous actin levels of spines in the ASMko treated neurons with 1α, 25-dihydroxyvitamin D3 or dexamethasone, respectively, as monitored by phalloidin staining (Fig 5E). We did not observe significant effects on filamentous actin in dendritic spines of similarly treated wt neurons (Supplementary Fig 4C). In all, these results further supported the role of SM and NSM in ASMko dendritic spine actin modulation and provided with a pharmacological strategy to revert spine abnormalities in the mouse model for NPA.

### Oral administration of dexamethasone reverts SM and RhoA synaptic anomalies, restores dendritic spine size, prevents neuronal death and improves functional deficits in ASMko females

**Figure 6:**
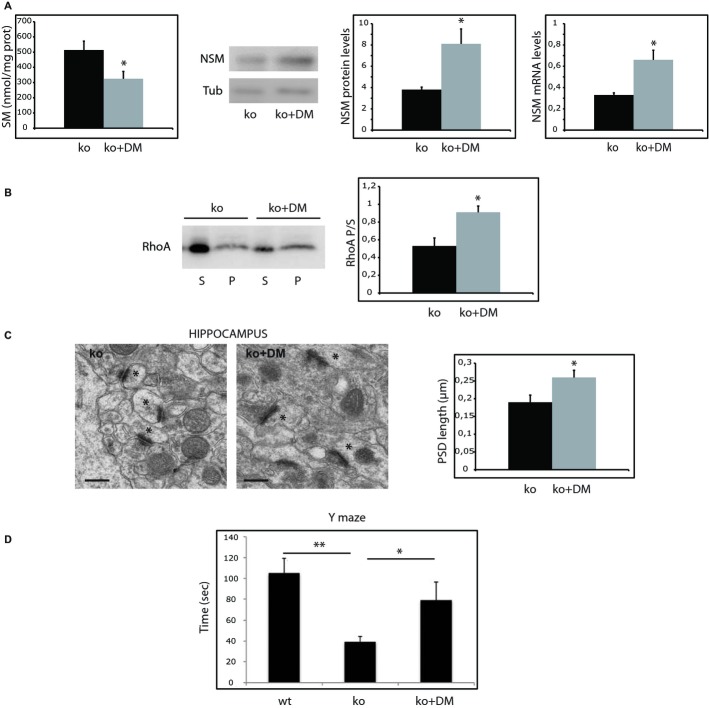
Oral treatment with dexamethasone increases brain NSM mRNA and protein levels and reverts molecular, morphological and functional alterations in ASMko females.
Mean ± s.d. of SM levels (nmol/mg protein) in synaptosomes from ASMko females treated or not with dexamethasone (*n* = 10; *P = 0.03). Western blot of NSM and tubulin levels in synaptosomes derived from ASMko females treated or not with dexamethasone. Graphs show mean ± s.d. of NSM protein (normalized to tubulin) and mRNA levels (*n* = 10, *P_NSM_ _prot_ = 0.024; *P_NSM_ _mRNA_ = 0.03).Western blots shows RhoA levels in supernatants (S) and pellets (*P*) after 100 000 g centrifugation of synaptosomes from ASMko females treated or not with dexamethasone. Graph shows mean ± s.d. of the RhoA ratio pellet/supernatant in synaptosomes from ASMko females treated or not with dexamethasone (*n* = 10, *P = 0.01).Electron micrographs of synapses in the hippocampal CA1 stratum radiatum of ASMko females treated or not with dexamethasone. Spines are indicated by asterisks. Graph shows mean ± s.d. of PSD length in μm (*n* = 70 synapses in each of 3 mice per condition, *P = 0.031).Results of the Y-maze test in wt, ASMko and dexamethasone ASMko treated females. Graph shows mean ± s.d. of the time (in seconds) spent by the mice in the novel arm (*n* = 7; **P_ko vs wt_ = 0.009, *P_DM__ko vs ko_ = 0.021).

Our next aim was to test the efficiency of the aforementioned treatments *in vivo*. Since dexamethasone showed more pronounced effects on the *in vitro* reversion of aberrant molecular phenotypes we chose to use this synthetic glucocorticoid, which is able to cross the brain blood barrier (Stumpf *et al*, 1989; Stumpf, 2012) and is currently used for the treatment of different human diseases (van de Beek *et al*, 2012; De Cassan *et al*, 2012; Kanwar *et al*, 2013). Treatments started immediately after weaning in 1-month old wt and ASMko mice. Dexamethasone dissolved in ethanol was added to the drinking water at a concentration that ensured the consumption per mouse of 0.3 μg/g/day, which is a dose utilized for long term treatment in pediatric patients. Wt and ASMko mice were divided by gender in groups of ten animals each. Non-treated males and females were given ethanol in their drinking water at the same concentration than the dexamethasone-treated mice (0.1% v/v). Treatments were followed for 2.5 months. At the end of this period mice were sacrificed and synaptosomes were obtained. Dexamethasone treatment of wt mice did not alter SM levels nor RhoA membrane binding in synaptosomes (Supplementary Fig 4D). Among the treated ASMko males 50% of them showed reduced SM levels compared with non-treated ASMko males but the average reduction was a non-significant 16% (Supplementary Fig 5A). Also non significant were the changes in NSM protein levels and the 1.3-fold increase in RhoA membrane attachment in synaptosomes of dexamethasone treated ASMko males (Supplementary Fig 5B and C). However, all treated ASMko females showed SM reduction at their synaptic membranes, which in average reached a significant 36.7% compared to non-treated ASMko females (Fig 6A). That SM reduction was driven by NSM *in vivo* was supported by the dexamethasone-induced 113% increase in the enzyme levels (Fig 6A). This was accompanied by the transcriptional upregulation of the enzyme, which mRNA levels were two-fold higher in brain extracts from dexamethasone treated ASMko females (Fig 6A). In turn, RhoA membrane binding was enhanced by 1.7-fold (Fig 6B). Electron microscopy analysis showed a significant 36% increase in the PSD length of synapses of the hippocampal CA1 region in the ASMko treated females (Fig 6C). To determine whether other neuropathological changes were improved by the treatment we monitored neuronal death in the cerebellum, which is a pathological hallmark in ASMko mice brains already at 3 months of age (Macauley *et al*, 2008). Dexamethasone treatment prevented Purkinje cell loss to a significant extent (59% increased in the number of cells per area unit) (Supplementary Fig 1B). Finally, we ought to determine whether dexamethasone effects resulted in functional improvement. The dendritic spine phenotype in the hippocampus moved us to monitor spatial memory governed by this brain areas using the Y-maze test (Cognato *et al*, 2010). The time spent in the novel arm, indicative of memory ability, was indeed 2.7-fold reduced in the ASMko females compared to wt (wt: 104 ± 10 s; ASMko: 38 ± 5 s). Dexamethasone treatment significantly increased this time by two-fold in ASMko females (79 ± 9 s) indicating improved spatial memory. Given the benefitial effects of dexamethasone observed in the cerebellum (Supplementary Fig 1B) and because ataxia is evident in ASMko mice at 4 months of age (Horinouchi *et al*, 1995; Macauley *et al*, 2008), we investigated the impact of dexamethasone treatment in ASMko female motor coordination using the vertical pole test (Ogawa *et al*, 1985). While 100% wt females completed the test in <50 s none of the ASMko females did it (Supplementary Fig 1C). Notably, 63% of dexamethasone treated ASMko females performed the test in <50 s. Analysis of the data in a cumulative frequency graph clearly showed the distribution of wt, ASMko treated and ASMko non-treated mice in three differentiated groups (Supplementary Fig 1C).

Altogether, the results obtained *in vivo* demonstrate the efficiency of oral treatment with dexamethasone to revert dendritic spine molecular and morphological anomalies, to prevent cerebellar neuronal death and to amelliorate behavioural deficits in ASMko mouse females.

## Discussion

The work presented here makes three main contributions: (i) describes the aberrant phenotype of dendritic spines in the ASMko mouse, which is a model for NPA; (ii) characterizes the molecular mechanism underlying this aberrant phenotype; (iii) provides with pharmacological strategies to revert the anomalies *in vitro* and *in vivo*. We believe these contributions help to understand the yet poorly characterised role of lipids in synapses and open new therapeutical venues for the currently untreatable NPA.

Our results identify an actin regulatory pathway in dendritic spines, which links a plasma membrane lipid (SM), its catabolic enzymes (ASM and NSM), neurotransmitter receptors (mGluRs type I) and a small GTPase (RhoA) and its effectors (ROCK and profilinIIa). This pathway highlights a previously unknown influence of SM and sphingomyelinases on the dendritic spine actin cytoskeleton. Alterations in this pathway result in the spine anomalies we found in mice lacking ASM. Although the loss of ASM has a similar effect in dendritic spines of neurons analyzed in different brain areas, i.e. reduction in their size, the extent of size reduction varies. In neurons of the cortex this is such that it leads to the disappearance of these membrane protrusions. The differences among neuronal populations might be due to variations in the time of exposure or sensitivity (i.e. basal levels of SM, robustness of compensatory mechanisms) to the increased SM levels. Further studies are required to address this issue.

We demonstrate that SM accumulation impairs the membrane binding and activation of the RhoA pathway, which plays a key role in dendritic spine actin polymerization (Schubert *et al*, 2006). Gross topological SM-induced alteration of the synaptic membrane or deficient addition of lipid moieties to RhoA (essential for the binding of RhoGTPases to the plasma membrane (Newman ' Magee, 1993), could hurdle RhoA membrane attachment. However, these possibilities would not explain the specificity of the binding impairment for RhoA. In fact, cdc42 and Rac are not altered despite they require similar processing for their membrane attachment than RhoA and would likely be affected by gross topological alterations. In contrast, interaction with mGluR1/5 has been shown only for RhoA (Schubert *et al*, 2006). We thus propose that the low levels of these receptors at the synaptic membrane could confer specificity to the deficient membrane binding observed for this GTPase. Our results indeed underscore the influence of SM on the levels of mGluR1/5 in synaptic membranes. Cholesterol-sphingolipid enriched domains, rafts, regulate the expression of these receptors at the neuronal surface (Francesconi *et al*, 2009). In addition, the absence of ASM alters neuronal raft composition and functionality as evidenced by the impaired endocytosis and altered distribution of raft components in polarized hippocampal neurons (Galvan *et al*, 2008). Hence, it is possible that alterations in raft lipid composition (i.e. increased SM levels) have an effect on the stability and internalization of mGluR1/5 at the ASMko synaptic membrane, therefore affecting RhoA membrane distribution. In support for the involvement of raft alterations in the aberrant molecular phenotype we observed that, although raft abundance does not appear to be significantly affected since the raft bona fide marker flotillin is not altered, the presence of RhoA in raft domains is reduced in ASMko synaptosomes compared to wt (Supplementary Fig 6).

A surprising conclusion arising from our work is the relevant influence that ASM has on the lipid and protein composition of synaptic membranes, despite being a lysosomal enzyme (Stoffel, 1999). However, the presence of a pool of ASM has been reported at the plasma membrane (Grassme *et al*, 2001) and we consistently observed high SM levels at this cellular site in ASMko cultured hippocampal neurons and in non-lysosomal membranes derived from ASMko mouse brain extracts (Galvan *et al*, 2008). Here we extend these findings and describe that ASM deficiency also affects the lipid composition of postsynaptic membranes. The question arises about how an enzyme with an acidic optimal working environment may function at the neutral environment of the plasma/synaptic membrane. Data showing the ability of ASM to degrade SM within LDL particles at physiological pH values (Schissel *et al*, 1998) and the possibility that acidified microenvironments may exist at the cell surface (Bourguignon *et al*, 2004; Steinert *et al*, 2008) could explain this apparent inconsistency. It is also important to note that a very limited activity of this enzyme (1–2%) appears to be sufficient to avoid the severe neurological symptoms of NPA patients. In fact, this range of ASM residual activity distinguishes between the type A and the non-neurological type B forms of Niemann Pick disease (Schuchman, 2009). Our findings indicate that although ASM is necessary for neuronal function little activity would be enough to fulfil its task. Thus, therapies for NPA aimed to increase sphingomyelinase activity at the plasma/synaptic membranes might be effective even with low efficiency.

Our results suggest that one such strategy is the enhancement of NSM activity. We report that two NSM activators, the active form of vitamin D and the glucocorticoid dexamethasone, have the ability to reduce the amount of SM by increasing NSM protein levels and activity at synapses and to rescue aberrant phenotypes *in vitro*. Although with low efficiency, both compounds cross the brain blood barrier (Pardridge *et al*, 1985; Stumpf *et al*, 1989; Stumpf, 2012), and have been already used for long-term treatment of different human diseases (Bonthius ' Karacay, 2002; Holick, 2005; Cole, 2006). We report that *in vivo* treatments by oral administration of dexamethasone to ASMko females significantly reduced synaptic SM and increased NSM protein levels, reverted aberrant synaptic molecular and morphological phenotypes, prevented neuronal degeneration and improved functional deficits. We found a similar tendency in dexamethasone treated males but the effects were not statistically significant. The different outcome between females and males might respond to a less efficient NSM enhancement in the later, in turn preventing SM reduction (compare Fig 6A and B with Supplementary Fig 5). Our results evidence that dexamethasone induces the transcriptional activation of the enzyme in the brains of ASMko treated females. The fact that we observe increased NSM levels upon *in vitro* treatment of isolated synaptosomes suggests that local transcription might be taking place. Further work will detailed how dexamethasone enhances NSM transcription. A likely possibility would involve glucocorticoid (GC) receptors, for which dexamethasone is an agonist and which expression levels are different in males and females and could explain the different response between genders. Our data in synaptosomal preparations and the recently reported presence of GC receptors in dendritic spines (Jafari *et al*, 2012), would support a direct effect of dexamethasone in synapses. Although basal or slightly high GC concentrations are needed for learning and memory processes, chronic excess in GC levels has adverse effects in the nervous system including atrophy of neuronal processes and disruption of plasticity (Sapolsky, 1999). This is in apparent contradiction with the positive effects we observe in dexamethasone treated ASMko mice and raises concern about the possible long-term exposure of ASMko neurons to this synthetic GC. However, expression of GC receptors at dendritic spines is increased by activation of mGluR type 1 (Jafari *et al*, 2012), which levels we find reduced in ASMko synapses. It might be that response to GC is chronically impaired in ASMko mice and that the long-term exposure to a GC receptor agonist restores this response to normal, not high, levels resulting in the improvement and not in the impairment of synaptic events.

Alternative to a direct effect, the influence of the orally administered dexamethasone in synaptic SM levels and function might be indirect through its immunomodulatory properties. While the majority of studies have emphasized the immunosuppressive role of GCs, immunoenhancement effects can occur through the differential modulation of cytokine levels (Wilckens, 1995). Indeed, while acute peritoneal dexamethasone administration resulted in reduced levels of cytokines in the injured hippocampus, dexamethasone treatment prior to injury increased cytokine expression including that of TNFα (Bruccoleri *et al*, 1999). There is increasing evidence that pretreatment with this cytokine may protect neurons against injuries (Figiel, 2008). Interestingly, TNFα is also a potent activator of NSM at synapses playing a role in neurotransmitter receptor clustering and synaptic plasticity (Wheeler *et al*, 2009). Therefore, a dexamethasone-induced increase of TNFα levels might account for the enhanced NSM activity and reduced SM levels at synapses of ASMko treated mice. It might thus be that TNFα exerts two positive actions in the ASMko brains: facilitating synaptic plasticity and preventing neuronal damage.

A third, not excluding, possibility would involve the anti-inflammatory effects of dexamethasone (Laste *et al*, 2013). To explore this possibility we treated ASMko synaptosomes or ASMko females with ibuprofen, a non-steroid anti-inflammatory drug (NSAID). Using the same protocols as for dexamethasone we did not see any difference in SM levels or RhoA membrane binding *in vitro* (Supplementary Fig 7A). Synaptosomes derived from ASMko females after oral administration of ibuprofen for 2.5 months showed a tendency for SM reduction and increased RhoA membrane binding (Supplementary Fig 7B). However, the differences were not statistically significant with respect to non treated mice. These results do not allow us to rule out that anti-inflammatory effects of dexamethasone are involved in the positive effects observed in the treated ASMko mice but suggest that, at least in the conditions tested, these effects are not sufficient to restore the normal phenotype. In any event, these results encourage research aimed to determine the potential benefits of the use of NSAIDs for NPA treatment.

Finally, the present results together with other recent reports (reviewed in Ledesma *et al*, 2011) stress the view that NPA should not be regarded simply as a lysosomal lipid storage disease. Sphingolipid alterations at the plasma and synaptic membranes likely contribute, as much or even more, to the neuronal pathology than the accumulation of these lipids in lysosomes. Therefore, therapies aimed to correct these alterations should be taken into account.

## Materials and Methods

### Materials

Antibodies against the following molecules were used for Western blots: RhoA (rabbit polyclonal 67B9 Cell Signaling Technology Inc., Danvers, MA, USA), ROCK (mouse monoclonal clone 21 BD transduction laboratories, Becton, Dickinson and Company, Franklin Lakes, NJ, USA), Profilin IIa (rabbit polyclonal, a gift from C.G. Dotti laboratory, CBMSO Madrid, Spain), alpha-tubulin (mouse monoclonal 7291; Abcam plc, Cambridge Science Park, Cambridge, UK), Synaptophysin (mouse monoclonal Boehringer), PSD95 (mouse monoclonal Upstate Biotechnology), mGluR1 (Rabbit polyclonal 445870; Calbiochem, EMD Millipore Corporation, Billerica, MA, USA), mGluR5 (Mouse monoclonal 5675; Millipore, EMD Millipore Corporation), NSM (mouse monoclonal sc-166637; Santa Cruz Biotechnology Inc., Dallas, TX, USA), Calbindin (rabbit polyclonal PC253L; Merck Millipore, Merck KGaA, Darmstadt, Germany). Goat anti-rabbit and Rabbit anti-mouse HRP-conjugated antibodies (Dakocytomation) were used as secondary antibodies.

### Mice

A breeding colony was established from a couple of ASM heterozygous C57BL/6 mice (Horinouchi *et al*, 1995), kindly donated by E.H. Schuchman (Mount Sinai School of Medicine, New York). Male littermates of 4–6 months of age wt and ASMko mice were compared. All procedures involving the use of animals were conducted according to guidelines specified for the animal protection and welfare by the Spanish Ministry of Agriculture.

### DiOlistic labeling of dendritic spines

For diOlistic imaging of dendritic spines, mice (four for each genotype) were anesthetized and perfused with 4% paraformaldehyde in phosphate buffer (PB, 0.1 M pH 7.4). The brains were postfixed, washed in PB and cut into 300-μm sagittal sections on a vibratome (Leica VT 1000S; Leica Microsystems GmbH, Wetzlar, Germany). Fluorescent labeling of brain sections was done according to a modified protocol of the original diOlistic labeling (Pavlowsky *et al*, 2010) described by Gan *et al* (2000). Briefly, tungsten particles coated with 1′-dioctadecyl-3,3,3′,3′-tetramethylindocarbocyanine perchlorate crystals (DiI) were propelled into brain sections from a distance of 0.5 cm using a biolistic ‘Helios gene gun system’ (BioRad Laboratories, Inc., Berkeley, CA, USA) at a pressure of 120 psi. A membrane filter with a 3.0 μm pore size (Millipore) was placed between the gun and the tissue to filter out large clusters of coated particles. After one single shot, slices were placed in 4% paraformaldehyde for 2 h, washed in PB and mounted on slides. A confocal microscope (Zeiss LSM-5 Pascal, Germany) was used to image the labeled structures. Optical sections were collected using a 40 × immersion oil objective with a digital zoom of 4 ×. At least 10 *z*-stack images consisting of 10–15 sections (512 × 512 pixels, 80–100-μm-long dendritic segments) spaced 0.5 μm apart were collected for each animal and for each area analyzed to generate the data set. Spine density was analyzed in CA1 neurons of the hippocampus and in pyramidal neurons of the S1 cortex. Dendritic segments and spines were analyzed quantitatively by using 3D image stacks using ImageJ software 1.34S (Wayne Rasband, National Institute of Health, Bethesda, MD, USA, public domain). All dendritic protrusions with a clearly recognizable neck connected to the shaft of the dendrite were counted as spines. Spine number and dendritic length were measured by projecting all the stacks of an image into a single plane (maximum projection) with the observer blind to the experimental conditions. The raw data obtained in ImageJ (Image processing and Analysis in Java, Developed by Wayne Rasband, National Institute of Health) were exported to Microsoft Excel for further analysis.

### Electron microscopy

Mice were anesthetized with an intraperitoneal injection of ketamine-xylazine 1:1 (0.1 ml/kg) and perfused through the left ventricle with a mixture of paraformaldehyde (4%) and glutaraldehyde (2%) in PB. Brains were postfixed in the same solution for 4 h. The dorsal hippocampus or the cerebellum were cut into transverse slabs that were postfixed in 1% osmium tetroxide (in 0.1 M cacodylate buffer), dehydrated in ethanol and embedded in Epon-Araldite. Serial ultrathin sections of the CA1 region or the molecular cerebellar layer were collected on pioloform-coated, single-hole grids, and stained with uranyl acetate and lead citrate. The sections were analysed with a JEM-1010 transmission electron microscope (Jeol, Japan) equipped with a side-mounted CCD camera Mega View III from Olympus Soft Imaging System GmBH (Muenster, Germany). Synapses were sampled randomly in the proximal part of stratum radiatum and photographed at a magnification of ×75.000 (∼70 synapses per mouse, *n* = 3 mice per group). The area of dendritic spines and the length of the PSD were measured with the AnalySIS software (Soft Imaging System GmBH).

### Synaptosomal isolation

The protocol has been well described in Schubert *et al* (2006) and is based on methods set by Cohen *et al* (1977) and Carlin *et al* (1980). Briefly, mouse brains were homogenized in buffer A (0.32 mM sucrose, 1 mM MgCl_2_, 0.5 mM CaCl_2_, 1 mM NaHCO_3_, protease inhibitors) and centrifuged at 1400 *g* for 10 min to obtain supernatant S1 and pellet P1. P1 was homogenized in buffer A and centrifuged at 700 *g* for 10 min. The resulting supernatant was combined with the previous S1 and centrifuged at 13 800 *g* for 10 min. The obtained supernatant (S2) was centrifuged at 17 000 *g* for 1 h. The resulting supernatant (S3) constitutes the cytosolic fraction. The resulting pellet resuspended in buffer B (0.32 mM sucrose, 1 mM NaHCO_3_, 1 mM EGTA, 1 mM dithiothreitol, protease inhibitors) is the crude synaptosomal fraction. To obtain the pure synaptosomal fraction, the crude fraction was loaded on a discontinuous sucrose gradient (1 and 1.4 M sucrose) and centrifuged for 65 min at 82,500 *g*. The pure synaptosomal fraction was recovered from the interphase between 1 and 1.4 M sucrose. To obtain a fraction enriched in postsynaptic membranes (PSD), the protein amount was calculated in the pure synaptosomal fraction, and a 4 mg/ml solution was prepared with buffer B. An equal volume of a solution composed of Triton X-100, 0.5 mM Hepes/KOH, and protease inhibitors was added and stirred for 15 min on ice. The sample was centrifuged at 28,000 *g* for 40 min to obtain supernatant LS1. LS1 was centrifuged at 165 000 *g* for 120 min to obtain pellet LP2. LP2 was then homogenized in buffer B and loaded onto a discontinuous sucrose density gradient composed of 1.0, 1.5, and 2.1 M sucrose and centrifuged at 201,800 *g* for 60 min. The PSD fraction was obtained from the interphase between the sample and 1.0 M sucrose.

### Lipid analysis

For the mass lipid analysis of postsynaptic enriched fractions (PSD) lipid extracts were prepared as described in Galvan *et al* (2008) and analyzed for phospholipids (organic phosphate) (Van Veldhoven ' Bell, 1988) or enzymatic quantification of cholesterol (Van Veldhoven *et al*, 1998). To quantify SM, lipid extracts were dried in presence of detergent (Thesit), and SM was subsequently converted into choline by means of sphingomyelinase, alkaline phosphatase, and coupled to the production of fluorescence with choline oxidase, peroxidase and homovanillic acid as modified from Hojjati and Jiang (2006) and optimized for extracts (Van Veldhoven P.P. and De Schryver E., unpublished data).

### Synaptosomal treatments

For stimulation crude synaptosomal fractions were incubated for 3 min at 37°C under gentle agitation with 5 mM KCl (control) or 55-mM KCl (stimulated). Reactions were stopped by placing the samples on ice.

To modulate SM levels in synaptosomes, freshly isolated pure synaptosomal fractions were incubated at 37°C for 1 h under gentle agitation either with 100 μg/ml SM (Sigma-Aldrich, Co., St. Louis, MO, USA) (added from a stock prepared in ethanol) to increase the lipid levels or with 0.1 μM 1α, 25-dihydroxyvitamin D3 (Sigma-Aldrich) or dexamethasone (Sigma-Aldrich) to decrease them. To analyze the effect of the treatments on the membrane attachment of RhoA and its effectors, treated and non treated samples were centrifuged at 100 000 g and 4°C for 1 h. Proteins in supernatants and pellets were resolved by SDS-PAGE and electroblotted to nitrocellulose membranes. These were incubated with specific primary antibodies and with peroxidase-linked secondary antibodies. Chemiluminescent signal in the Western blot was detected by ECL (GE Healthcare Co., Fairfield, CT, USA) and quantified under non saturated conditions using a densitometer and the software Quantity One.

### Hippocampal neuronal cultures and treatments

Primary cultures of hippocampal neurons were prepared from wt and ASMko mice brain embryos as described in Goslin ' Banker (1991). For our experiments hippocampal neurons were kept in culture for 15 days or more when they have reached full synapse maturation. SM levels were modulated by several means: (i) addition to wt neurons of 40 μM SM (Sigma-Aldrich), which was added from a stock prepared in ethanol that ensured a final ethanol concentration of less that 1% in the neuronal medium to avoid toxicity. Same amount of ethanol without SM was added to control neuronal cultures; (ii) incubation of ASMko neurons with *Bacillus aureus* Smase (Sigma-Aldrich) at 0.1 unit/100 μl medium (as indicated in Galvan *et al*, 2008). For the activation of Neutral sphingomyelinase 1α, 25-dihydroxyvitamin D3 or dexamethasone were added everyday to the culture medium at a final concentration of 0.1 μM, starting at 9DIV until 15 DIV. To determine the amount of filamentous actin in all the aforementioned experiments, neurons were fixed in PFA/SEM buffer (4% paraformaldehyde, 0.12 M sucrose, 2 mM EGTA and 2 mM MgCl_2_ in PBS) for 10 min, quenched with 50 mM ammoniun chloride and extracted with 0.1% Triton X-100 at RT. Filamentous actin was labelled by incubation with TRITC-conjugated phalloidin (Sigma-Aldrich) as in Schubert *et al* (2006). Samples were analyzed in a Leica fluorescence microscope. Phalloidin associated fluorescence was quantitated in dendritic spines identified, by triple labelling immunofluorescence, as PSD95 positive protrusions derived from the dendritic shaft, which was labelled with MAP2. Pixel intensity was determined with ImageJ software. Mean intensity in spines was calculated per area unit.

### Rhotekin binding assay

The EZ-detect™ Rho Activation kit (Pierce Protein Biology Products; Themo Fisher Scientific Inc., Rockford, IL, USA) was used to determine the affinity of RhoA to its downstream effector Rhotekin and thus its activity. Fresh crude synaptosome preparations from age-matched wt and ASMko mice containing 500 μg of protein were processed, in parallel, following the manufacturers instructions. The resulting samples were analyzed by Western blot using an antibody against RhoA.

### Raft isolation

Synaptosomes were incubated in TNE buffer (100 mM Tris, 2 mM NaCl, 10 mM EDTA, pH 7.4) containing 0.5% Triton X-114 and protease inhibitor cocktail (complete EDTA-free; Roche, Basel, Switzerland)(40 min, 4°C), then brought to 60% sucrose. A 35 and 5% sucrose step gradient in TNE was overlaid on samples and ultra centrifuged (19 h, 73 000 *g*). After centrifugation, 13 fractions of 1 ml each were collected from top to bottom of the tube. Detergent-insoluble material (rafts) was obtained in the lighter fractions (1–7).

### Immunoprecipitation

Synaptosomal preparations were precleared with G-Sepharose beads and incubated or not with 3 μg anti-RhoA antibody for 1 h at 4°C. Subsequently, protein G-Sepharose beads were added and samples were incubated overnight at 4°C under gentle rotation. Samples were then washed twice (20 min each washing) with immunoprecipitation buffer (1% Triton X-100, 100 mM NaCl, 2 mM EDTA, 10 mM Tris–HCl, 1 mM Na3VO4, pH 7.5 and protease inhibitors), twice with high salt buffer (same as immunoprecipitation buffer but with 500 mM NaCL and no Triton X-100) and once with low salt buffer (same as immunoprecipitation buffer but without NaCl and TritonX-100). Beads were pelleted in between washes by centrifugation at 1600 *g* for 30 s. After the final wash, beads were pelleted down by high-speed centrifugation and the immunocomplexes released from beads and analyzed by Western blot using anti-mGluR1 or anti-mGluR5 antibodies.

### Oral treatment with dexamethasone and ibuprofen

Wt and ASMko mice were divided by gender in groups of ten animals each. Treatments started immediately after weaning when mice were 1 month old. Dexamethasone dissolved in ethanol or ibuprofen (pirexin solution, Juventus laboratories) was added to the drinking water at a concentration of 1.5 μg/ml and 1 mg/ml (as in Ezell *et al*, 2012), respectively. Considering that the regular daily consumption of water per mice is 4 ml and the average weight is 20 g this concentration ensured the consumption per mouse of approximately 0.3 μg/g/day dexamethasone (ethanol consumption was lower than 5 μl/mouse/day). Non-treated males and females were given ethanol in their drinking water at the same concentration than the dexamethasone-treated mice. The drinking water with or without dexamethasone or ibuprofen was renewed every 3 days. Treatments went on for 2.5 months. At this time point mice were evaluated in behavioural tests (see below). They were subsequently sacrificed for synaptoisolation from their brains and biochemical analysis.

### Measurement of NSM mRNA

Total RNA from brain cortex homogenates was obtained by Trizol Reagent (Ambion /RNA. Life Technologies Co., Grand Island, NY, USA) and chloroform extraction. RNA was further cleaned up using Rneasy Mini kit (Qiagen, Hilden, Germany). RNA concentration was estimated by absorbance at 260 nm using a Nanodrop ND-100 (Thermoscientific; Themo Fisher Scientific Inc.). The retrotranscription to first strand cDNA war performed using RevertAid H Minus First Strand c DNA Synthesis kit from Thermo Scientific. qPCR was performed using GoTaq® qPCR Master Mix (Promega Co., Madison, WI, USA) and ABI PRISM 7900HT SDS (Applied Biosystems; Life Technologies Co.). For the detection of NSM2 transcripts the following primers (Sigma-Aldrich) were used: Nsm2_fw: 5^′^-TGCTGGACACAAACGGTCT; Nsm2_rev: 5′ – GTTGTCCGGGGTACACACAT. The three housekeeping genes GAPDH, GUSB and HPRT1 were used as endogenous controls.

### NSM activity

NSM activity was measured in synaptosomal extracts with the fluorimetric kit from Cayman Chemical Company (Sphingomyelinase Flourimetric assay kit, 10006964). Resorufin fluorescence was analyzed using the fluorometer FLUOstar OPTIMA from BMG LABTECH GmbH (Ortenberg, Germany).

### Neuronal death

Neuronal death was monitored in Purkinje cells of ASMko dexamethasomne treated or non treated females by immunofluorescence of brain tissue using an antibody against calbindin, which is a specific marker for these neurons. Images were obtained as *Z*-stacks using a confocal LSM 510 Meta coupled to a microscope Axiovert 200 (Zeiss). Number of calbindin positive cells were counted per area unit using the Image JA 1.45b software.

### Behavioural tests

The Y maze test was performed as in Cognato *et al* (2010). Briefly, during a first trial (training, 5 min), mice were allowed to explore only two arms (start and the other arm) with the third arm (novel arm) closed. For the second trial, mice were placed back in the same starting arm, with free access to all three arms for 5 min. The time spent in the novel arm was counted. The vertical pole test was performed as previously described (Ogawa *et al*, 1985). Briefly, mice were placed head-downward at the top of a vertical rough-surfaced pole (diameter 8 mm; height 55 cm) and let descend in a round of habituation. Then, mice were placed head-upward at the top of the pole. The total time until they descended to the floor was recorded with a maximum duration of 190 s. Ten age-matched wt, ASMko or dexamethasone-treated ASMko females were evaluated. Mice that did not move from the top of the pole after 190 s were not scored.

### Statistical analysis

Student's *t*-test and one-way ANOVA were used for statistical analysis of the data. *P* values lower than 0.05 were considered significant. In the figures asterisks indicate *P* values as follows: *< 0.05; **< 0.02; ***< 0.001. For the analysis of motor coordination after dexamethasone treatment the chi-squared test was utilized. *P* values lower than 0.05 were considered significant.

**The paper explained**

**Problem**

Although lipids are increasingly well recognized as key players in synaptic function, little is known about the molecular basis of their involvement. This information is essential to understand the etiology of the many lipidoses leading to cognitive impairment, which currently have poor prognosis. Niemann Pick disease type A (NPA) is an untreatable sphingolipidosis caused by loss of function mutations in the acid sphingomyelinase (ASM) gene leading to cellular sphingomyelin (SM) accumulation, severe mental retardation and death in early childhood. Although therapeutical strategies aimed at reducing SM levels have been tested in mice lacking ASM, which mimic the disease, the impact on brain pathology has been limited.

**Results**

We show that high SM levels at synapses of sphingomyelinase knock out mice (ASMko) diminish dendritic spine number and size by reducing filamentous actin. The molecular mechanism underlying these defects involves reduction of group 1 metabotropic glutamate receptors levels, which impairs the binding of the small GTPase RhoA to the postsynaptic membrane and the activation of its downstream effectors RockII and profilinIIa. Pharmacological activators (Vitamin D3 and dexamethasone) of the neutral sphingomyelinase reduce the levels of synaptic SM and restore RhoA membrane binding and filamentous actin levels *in vitro*. Oral treatment with dexamethasone causes similar effects in ASMko females by restoring dendritic spine size, preventing neuronal damage and leading to functional improvement.

**Impact**

Our study identifies a novel pathway by which a lipid (SM) and its catabolic enzymes modulate actin cytoskeleton in dendritic spines. We describe the alterations of this pathway in a mouse model for NPA and prove the efficiency of a pharmacological strategy to revert these alterations *in vitro* and *in vivo*. The fact that this strategy is based on the oral administration of dexamethasone, a compound that crosses the brain blood barrier and is already used for long-term treatments in different human diseases, enhances the possibilities of clinical applicability to NPA patients. Importantly, our findings could be relevant for patients with neurological disorders other than NPA that also exhibit aberrant SM accumulation.
