# Supplementary material for: Pharmacological reversion of sphingomyelin-induced dendritic spine anomalies in a Niemann Pick disease type A mouse model
Source: EMBO Mol Med. 2014 Jan 21;6(3):398–413. doi: 10.1002/emmm.201302649 (PMC3958313; doi:10.1002/emmm.201302649)
Supplement: Supplementary file 1 [file emmm0006-0398-sd1.pdf]

Pharmacological reversion of sphingomyelin-induced dendritic spine anomalies in a mouse model for Niemann Pick disease type A

Ana I. Arroyo, Paola G. Camoletto, Laura Morando, Marco Sassoe-Pognetto, Maurizio Giustetto, Paul P. Van Veldhoven, Edward H. Schuchman and Maria Dolores Ledesma

Corresponding author: Maria Dolores Ledesma, Centro de Biologia Molecular Severo Ochoa

Review timeline:

Submission date:	18 February 2013
Editorial Decision:	28 August 2012
Resubmission:	18 February 2013
Editorial Decision:	12 March 2013
Revision received:	16 October 2013
Editorial Decision:	31 October 2013
Revision received:	20 November 2013
Accepted:	28 November 2013

Transaction Report:

(Note: With the exception of the correction of typographical or spelling errors that could be a source of ambiguity, letters and reports are not edited. The original formatting of letters and referee reports may not be reflected in this compilation.)

Editor: Natascha Bushati / Roberto Buccione

1st Editorial Decision 28 August 2012

Thank you for the submission of your manuscript "Role of sphingomyelin on dendritic spine actin cytoskeletal anomalies and their pharmacological reversion in Type A Niemann-Pick disease" and please accept my apologies for not replying earlier. I have now had the opportunity to carefully read your paper and the related literature and I have also discussed it with my colleagues. I am afraid that we concluded that the manuscript is not well suited for publication in EMBO Molecular Medicine and have therefore decided not to proceed with peer review.

The manuscript reports that in hippocampal neurons of mice lacking ASM (ASMko), reduced RhoA membrane targeting and activity lead to lower levels of ROCK and profilin and subsequently actin cytoskeletal defects, which cause a pronounced reduction in dendritic spine size. You show that dendritic spine size and phalloidin labelling are severely reduced in ASMko hippocampal neurons, which can be mimicked by addition of SM to wt neurons and can be rescued by expression of Smase in ASMko cultures. Using wt and ASMko-derived synaptosomes highly enriched in postsynaptic membranes, you show that the SM content in ASMko synaptosomes is increased by 2-fold. Moreover, total, bound and active RhoA, ROCK and profilinIIa and mGluR1/5 are all significantly reduced in ASMko synaptosomes, which can be mimicked by addition of SM to wt synaptosomes. You demonstrate that upon stimulation, mGluR1/5 interaction with RhoA and susequently RhoA activity is not enhanced in ASMko conditions. Incubations of ASMko synaptosomes with the active form of Vitamin D3 or DM lead to a reduction in SM levels and an increase in RhoA membrane binding and to restoration of filamentous actin levels.

We appreciate that you show a link between levels of glutamate receptors, RhoA activity, and the actin cytoskeletal defects in ASMko neurons. However, we are not persuaded that the manuscript provides the sort of pathophysiological relevance and conceptual advance we would expect in an EMBO Molecular Medicine article. Specifically, it remains unknown whether the suggested therapeutic approach indeed results in functional improvement in an appropriate in vivo system.

I am sorry that I could not bring better news this time but hope that this negative decision does not prevent you from considering our journal for the publication of your future studies.

Resubmission 18 February 2013

A few months ago we submitted a manuscript to EMBO Molecular Medicine in which we described dendritic spine anomalies in a mouse model for Niemann Pick disease type A. We also characterized the molecular mechanism underlying these alterations identifying a novel regulatory pathway for actin cytoskeleton in the spines. This pathway involves sphingomyelin, its catabolic enzymes, group 1 metabotropic glutamate receptors and the small GTPase RhoA and its effectors RockII and profilinIIa. In addition, we reported the success of the pharmacological activation of neutral sphingomyelinase to revert the spine anomalies in vitro. At that time your decision was not to proceed with peer review, as it remained unknown whether the suggested therapeutic approach resulted in functional improvement in an appropriate in vivo system. Nonetheless, you declared no objection to consider a new manuscript on the same topic provided we obtained the abovementioned data. Encouraged by your criticism and comment we have tested the efficiency of the pharmacological strategy in the mouse model for NPA. We now report that oral treatment with dexamethasone, a compound that crosses the brain blood barrier and is approved for human use, not only reverts the aberrant molecular phenotype in dendritic spines but also improves functional deficits in the treated mice. These results suggest that glucocorticoid-based therapies could be applicable to NPA. We address this possibility in the discussion.
Because our work describes a novel physiological and pathological role for a lipid at synapses, offers mechanistic insight, and presents in vivo evidence that opens therapeutical perspectives for an untreatable disease, I hope you can now consider it

2nd Editorial Decision 12 March 2013

Thank you for the submission of your manuscript to EMBO Molecular Medicine. We have now received reports from the three Reviewers whom we asked to evaluate your manuscript

You will see that while two Reviewers are critical but generally supportive, one Reviewer is quite negative. In general, important points are raised that question the impact and conclusiveness of the results, thus preventing us from considering publication at this time.

I feel it unnecessary to list all the points in detail as they are well illustrated by the Reviewers. On the whole, the main concerns raised by all three Reviewers are that 1) the mechanism through which dexamethasone reduces sphingomyelin and improves dendritic spine pathology needs to addressed and 2) the quality of the data needs to be considerably improved (including additional controls).

While publication of the paper cannot be considered at this stage, we would be prepared to consider a suitably revised submission, with the understanding that all Reviewers' concerns must be fully addressed with additional experimental data where appropriate and that acceptance of the manuscript will entail a second round of review. I am prepared, provided you comply with the above requirements, to overlook Reviewer 2's negative stance, but not his/her well-taken items of concern.

Please note that it is EMBO Molecular Medicine policy to allow a single round of revision only and that, therefore, acceptance or rejection of the manuscript will depend on the completeness of your responses included in the next, final version of the manuscript.

Since the required revision in this case appears to require a significant amount of time, additional work and experimentation and might be technically challenging, I would therefore understand if you chose to rather seek publication elsewhere at this stage. Should you do so, we would welcome a message to this effect.

As you know, EMBO Molecular Medicine has a "scooping protection" policy, whereby similar findings that are published by others during review or revision are not a criterion for rejection. However, I do ask you to get in touch with us after three months if you have not completed your revision, to update us on the status. Please also contact us as soon as possible if similar work is published elsewhere.

I look forward to seeing a revised form of your manuscript as soon as possible.

***** Reviewer's comments *****

Referee #1 (Remarks):

This manuscript examines the effect of sphingomyelin (SM) as a modulator of the dendritic spine actin cytoskeleton. The authors show that the increased SM levels in neurons from ASMko mice is associated with reduced spine number, and impair attachment of cytoskeletal attachments to the synaptic membrane. Pharmacological reduction of SM levels can rescue the ASMko dendritic spine phenotype in vitro, and improve neurological function in vivo. These studies have implications for possible approaches for NPA disease.

The observation that SM levels affect dendritic spine formation and attachment to the actin cytoskeleton is an important finding, and sheds light on the etiology of the neuropathological abnormalities in NPA disease. In addition, the pharmacological approach using dexamethasone to phenotypically correct the SM excess and dendritic spine phenotype has potential as a therapeutic. Less clear is the mechanism through which dexamethasone is reducing SM levels in vitro, and the mechanism through which it is acting in vivo. The authors suggest the activation of neutral sphingomylinase (NSM) is responsible, but do not provide data to support this claim. Since glucocorticoids can have diverse cellular effects, through both transcriptional and non-genomic actions, further experiments are needed to clarify the mechanism responsible for the phenotypic correction. There are additional concerns related to data and need to show controls.

Specific concerns:

1. The authors propose that dex acts through activation of NSM. However, no data is presented demonstrating either increased NSM protein levels or activity upon dex stimulation. Fig 5A shows that there is no difference in NSM levels between WT and KO cells. What does dex treatment do to NSM levels or activity in KO cells? If NSM is not increased, then are SM levels being reduced by an alternative mechanism (e.g., increased availability/delivery of SM to the site of NSM)?

2. The image quality of the epifluorescence images for the phalloidin staining presented in multiple figures is marginal, such that specificity and identification of actin-membrane contacts are difficult to appreciate. In addition, the text indicates that co-staining was performed with the post-synaptic marker PD95 (Fig. 1C), but staining is not shown.

3. For the SM addition experiments it is unclear how and in what vehicle the SM was added to the cells. Vehicle only controls are needed.

4. In Fig. 3C, the cytoskeletal proteins are characterized with respect to shift from pellet after SM treatment. The significance of the shift - presumably from a membrane-bound to non-membrane-bound location - needs to be discussed in the Results. It would seem that perhaps a more relevant comparison would be treatment of KO with sphingomyelinase.

5. The TLC data presented in Fig. 5B is not convincing. Was the data in graph derived from triplicate values? MS-based determination of lipids is more quantitative and the accepted standard.

WT controls are needed for panels C and D.

6. The effect of dex treatment on the mice is intriguing, but the mechanism is unclear, as dex can have widespread effects in vivo. The data raises many questions. For example, are there neuropathological changes or reductions of SM in neurons that are improved with treatment? Is NSM transcriptionally upregulated or is activity increased with treatment? Is it possible that dex is working as an anti-inflammatory, and can similar effects be achieved, for example, using NSAIDs?

Minor concerns:
1. In Fig. S1A, the graphs labels are illegible
2. In Fig. 4C, it is unclear what comparisons are being made to state that RhoA activity is increased compared to control. Where is this data?
3. Pg. 7. The phrase "almost double" is awkward and probably unnecessary
4. Pg. 9. The word "enzyme" has been omitted between "main" and "responsible"

Referee #2 (Remarks):

This paper describes the contribution of sphingomyelin (SM) in the dendritic spine physiology in a mouse model of Niemann Pick disease type A (NPA). In particular, the paper attempts to provide a mechanistic link between abnormal levels of SM in the post-synaptic membrane and the altered morphology of dendrite spines in NPA. A pharmacological intervention based on the use of synthetic glucocorticoids is also proposed.

Although the role of lipids in spine dendrite physiology and how deregulation of these processes is involved in neurodegenerative disorders such as NPA are issues potentially interesting and therapeutically relevant, this work from Ledesma's lab does not provide enough insights into the mechanisms underlying these processes. Moreover, the set of data are not enough solid and convincing to support the conclusions made by the authors (see below for specific comments).

Specific comments:

Major points:
-Fig. 1. The data on altered dendritic spine number and morphology in the brain of ASMko mice are only showed in the layer 1 of S1 cortex and CA1 hippocampal pyramidal neurons. These data are very limited and they look enough convincing only in the cortex but appear not sufficiently convincing in the hippocampus. Why the hippocampus is less affected? What about the other brain regions?

-The role of Rho GTPases and their effectors as modulators of dendritic spine dynamics has been demonstrated in other works. However, in the present work there are not functional evidence demonstrating that SM increased levels in the post-synaptic membranes is causing synaptic spines alteration through the lost of normal synaptic protein levels of Rho GTPases and their effectors.

-By a mechanistic point of view a possible involvement of lipid-rafts is SM-mediated alteration of dendrites spine physiology is only speculated in the discussion. Direct evidence in this direction should be provided

-The authors used synthetic glucocorticoids in attempt to demonstrate a rescue of normal SM post-synaptic levels, Rho membrane binding and behavioral phenotype. The results are not convincing at all. There are no evidence that dexamethasone is specifically acting on SM levels in postsynaptic membranes og treated mice. The SM reduction in the brain of treated mice is very small, the increased membrane binding of Rho is even smaller. The behavioral phenotype was monitored by only vertical pole test that showed a very limited improved coordination in only 63% of treated females. More then one behavioral test should be used. Why males did not respond to the treatment is not sufficiently discussed. Then, most of the references cited are very old (Okazaki et al., 1989; Ramachandran et al., 1990; Stumpf et al 1989), these references have shown reducing SM levels

only in non neuronal cells and claim that synthetic glucocorticoids cross the BBB with very low efficiency.

- Many experiments lack of the necessary controls (loading controls in the coIPs experiments in fig 4, treatment of wild-type both cells and mice with glucocorticoid drugs in figs 5 and 6).

Minor points:
-In many figures key information are missing. This results in a difficult reading of the data. For example: Fig 1 B, which is the brain region analyzed? Fig 2 A, in the quantification of lipids which synaptosomal fraction has been analyzed?
-Fig 1C. The MAP2 staining cannot be distinguished. The images are of poor quality. Moreover, the small decrease in the fluorescent intensity (phalloidin) displayed in the graph is not representative of the reduction that can be detected by phalloidin staining in the fluorescence microscopy
Fig. 3B. Tubulin is not the best loading control for membrane fractions
Fig. 6. Again the panels in the figure lack of details that make the figure hard to read
Pag. 11. The authors claim that dexamethasone is currently prescribed for oral treatment of human diseases, key references lacking

Referee #3 (Comments on Novelty/Model System):

This work uses acid sphingomyelinase knockout mice, primary neurons in culture and synaptosomal preparations from wild type and mutant mice. These are excellent models to study mechanisms underlying neuronal dysfunction in Niemann Pick type A disease.

Referee #3 (Remarks):

Arroyo et al study the effects of sphingomyelin on dendritic spine morphology in models of Niemann Pick type A disease, a disorder characterized by acid sphingomyelinase deficiency (ASM). Using ASM knockout mice, primary neuronal cultures and synaptosome preparations, the authors show that increased sphingomyelin levels reduce dendritic spine numbers and size, lower levels of filamentous actin, decrease levels of type I glutamate receptors in synaptosome preparations, and impair membrane attachment of Rho A and its effectors. These analyses provide interesting new insights into the effects of sphinomyelin on dendritic spine morphology and may indicate a mechanism underlying neuronal dysfunction in disease. Pharmacologic activation of neutral sphingomyelinase is shown to rescue the dendritic phenotype of neurons in culture and improve functional deficits in ASM knockout mice. Since phenotypic rescue in vivo is accomplished by dexamethasone treatment, a manipulation that has many effects in the CNS, the authors should demonstrate that treatment reverses the dendritic spine defects shown in panel 1A. The status of additional pathologies present in the brains of these animals at the end of treatment, such as neuron loss, should be commented upon. A few other points should be addressed to strengthen the report.

What accounts for the decrease of mGluR levels in wt synaptosomes after treatment with SM (fig 4B)? Is there another fraction to which the receptors have translocated?

In fig 6B, it's curious that DM treatment seems to have its greatest effect on RhoA levels in the supernatant fraction rather than the pellet. Why is this? Is there a significant difference when one analyzes RhoA only in the pelleted fraction after DM treatment?

Loading controls are missing from a couple of western blots (figures 2a, 3Ba).

In Fig 4, panel 4Cc is referred to as panel 4D in the legend.

1st Revision - authors' response 16 October 2013

ANSWERS TO REVIEWER 1

We thank this reviewer for considering that our observations are important findings with potential therapeutical value. All his/her suggestions, which have helped us to clarify the mechanism for phenotypic correction, have been addressed as follows:

POINT 1. *The authors propose that dex acts through activation of NSM. However, no data is presented demonstrating either increased NSM protein levels or activity upon dex stimulation. Fig 5A shows that there is no difference in NSM levels between WT and KO cells. What does dex treatment do to NSM levels or activity in KO cells? If NSM is not increased, then are SM levels being reduced by an alternative mechanism (e.g., increased availability/delivery of SM to the site of NSM)?*

We have measured NSM protein levels and activity after dexamethasone treatment *in vitro* and *in vivo*. We find 30% and 15% increases in NSM protein levels and activity, respectively, in ASMko synaptosomes treated with dexamethasone (page 10, Figure 5C). The increase in NSM protein levels was even more pronounced (113%) in synaptosomes derived from ASMko females to which dexamethasone was orally administered for 2.5 months (page 12, Figure 6A). These results confirm that dexamethasone acts through enhancing the expression and therefore activity of NSM.

POINT 2. *The image quality of the epifluorescence images for the phalloidin staining presented in multiple figures is marginal, such that specificity and identification of actin-membrane contacts are difficult to appreciate. In addition, the text indicates that co-staining was performed with the post-synaptic marker PD95 (Fig. 1C), but staining is not shown.*

We now include, in all epifluorescence analysis for phalloidin staining, better quality images that also show the co-staining with the dendritic marker MAP2 and the postsynaptic marker PSD95 (new figures 1C, 2B and C, 5E and supplementary figure AC). Phalloidin associated fluorescence was quantitated in dendritic spines identified, by triple labelling immunofluorescence, as PSD95 positive protrusions derived from the dendritic shaft, which was labelled with MAP2. Mean intensity in spines was calculated per area unit. This is now explained in methods (page 23).

POINT 3. *For the SM addition experiments it is unclear how and in what vehicle the SM was added to the cells. Vehicle only controls are needed.*

We apologize for not having clarified this matter. The stock of SM was prepared in ethanol. For our experiments aliquots of this stock were added to reach a final SM concentration of 100mg/ml in synaptosomes or 40mM in cultured neurons. This results in less than 1% ethanol in the neuronal media to avoid toxicity. Same amount of ethanol was added to control experiments without SM. This is now better described in Methods (page 22).

POINT 4. *In Fig. 3C, the cytoskeletal proteins are characterized with respect to shift from pellet after SM treatment. The significance of the shift - presumably from a membrane-bound to non-membrane-bound location - needs to be discussed in the Results. It would seem that perhaps a more relevant comparison would be treatment of KO with sphingomyelinase.*

We now discuss the significance of the shift, which indeed reflects a differential binding to the membrane (page 7-8). Following this reviewer suggestion we have also compared RhoA behaviour after addition of sphingomyelinase to ASMko synaptosomes. This treatment increases RhoA membrane binding (page 8, Supplementary Figure C), consistent with the decrease observed upon addition of SM (Figure 3C).

POINT 5. *The TLC data presented in Fig. 5B is not convincing. Was the data in graph derived from triplicate values? MS-based determination of lipids is more quantitative and the accepted standard.*

We now provide with a more accurate measurement of SM levels by mass analysis, which allow as determining nmol/mg protein of SM. The data in the new graph derive from triplicate values (new Figure 5B).

WT controls are needed for panels C and D (now D and E).

We now show the effects of Vitamin D3 and dexamethasone treatments in wt synaptosomes and cultured neurons (Supplementary Figure D). These treatments had no significant effects on SM levels or RhoA membrane binding in wt synaptosomes and did not alter phalloidin levels in the spines of wt neurons. These results are summarized in page 10.

POINT 6. *The effect of dex treatment on the mice is intriguing, but the mechanism is unclear, as dex can have widespread effects in vivo. The data raises many questions. For example, are there neuropathological changes or reductions of SM in neurons that are improved with treatment?*

We thank this reviewer for this suggestion that has led to widen our knowledge on the effects of dexamethasone treatment *in vivo*. We have monitored neuronal death in the cerebellum, which is a pathological hallmark in ASMko mice brains already at three months of age (Horinouchi et al., 1995; Macauley et al., 2008). Dexamethasone treatment enhanced Purkinje cell survival to a significant extent (59%) (page 12, Supplementary figure AB).

Is NSM transcriptionally upregulated or is activity increased with treatment?

To address this question we have measured NSM mRNA in brain extracts of ASMko females treated or not with dexamethasone. We observed a significant 2-fold increase upon treatment, which could account for the 113% higher protein levels of NSM (page 12, Figure 6A). These results evidence the transcriptional upregulation of NSM by dexamethasone (discussed in page 16).

Is it possible that dex is working as an anti-inflammatory, and can similar effects be achieved, for example, using NSAIDs?

We have addressed this question *in vitro* and *in vivo*. Treatment of ASMko synaptosomes with the NSAID ibuprofen did not show alterations in SM levels or RhoA membrane binding (Supplementary Figure GA). Synaptosomes derived from ASMko females orally treated with ibuprofen for 2.5 months (using similar protocol of administration as for dexamethasone described in page 25) showed a tendency for SM reduction and increased RhoA membrane binding (Supplementary Figure GB). However, the differences were not statistically significant with respect to non treated females. These results do not allow us to rule out that anti inflammatory effects of dexamethasone contribute to the positive effects observed in the treated ASMko mice but suggest that, at least in the conditions tested, these effects are not sufficient to restore the normal phenotype (discussed in page 17). In any event this reviewer suggestion encourage us to analyse further the potential benefits of NSAIDs for NPA patients.

Minor concerns:

1.In Fig. S1A, the graphs labels are illegible.

Corrected

2. *In Fig. 4Cc, it is unclear what comparisons are being made to state that RhoA activity is increased compared to control. Where is this data?*

We apologize for not having explained sufficiently the data in this figure. The comparison made refers to the ability to upregulate RhoA activity following stimulation in ASMko synaptosomes compared to wt. Therefore, the values to be compared are those obtained in stimulated *vs* non stimulated wt samples <u>with</u> those obtained in stimulated *vs* non stimulated ASMko samples. We cannot compare non stimulated wt and ko samples between them since these samples were not controlled for the same amount of protein in these experiments. This is now better explained in page 9.

3. *Pg. 7. The phrase "almost double" is awkward and probably unnecessary.*

Corrected

4. *Pg. 9. The word "enzyme" has been omitted between "main" and "responsible".*

Corrected

ANSWERS TO REVIEWER 2

We thank this reviewer for appreciating the interest and potential therapeutical relevance of understanding the role of lipids in spine dendrite physiology and their deregulation. We are also grateful for his/her suggestions that have improved our work. They have been addressed as follows:

Major points:

POINT 1. *-Fig. 1. The data on altered dendritic spine number and morphology in the brain of ASMko mice are only showed in the layer 1 of S1 cortex and CA1 hippocampal pyramidal neurons. These data are very limited and they look enough convincing only in the cortex but appear not sufficiently convincing in the hippocampus. Why the hippocampus is less affected? What about the other brain regions?*

We chose to analyze cortex and hippocampus since these brain areas are most related to memory and learning abilities, which are affected in Niemann Pick disease type A patients. We reported that the reduction in the number of spines was only significant in the cortex (1.6-fold) and not in the hippocampus (page 5, Figure 1A). However, in this area we did observe a significant reduction in the spine size (1.7-fold) (page 5, Figure 1B). This is now better explained in the figure legend and also by labelling the panels in Figures 1A and B. To further satisfy this reviewer concern we have now quantified spine size in the cerebellum of ASMko mice where the reduction is also significant compared to wt (page 5, Supplementary figure AA). Altogether, these results show that the absence of ASM has a broad impact in dendritic spines of different neuronal populations ranging from spine loss in the cortex to reduced size in the hippocampus and cerebellum. The different impact might be due to the sensibility to SM level variations of the different neuronal populations.

POINT 2. *-The role of Rho GTPases and their effectors as modulators of dendritic spine dynamics has been demonstrated in other works. However, in the present work there are not functional evidence demonstrating that SM increased levels in the post-synaptic membranes is causing synaptic spines alteration through the lost of normal synaptic protein levels of Rho GTPases and their effectors.*

We now provide with additional evidence that high SM levels affect the RhoA pathway in ASMko synapses. Hence, we show that sphingomyelinase treatment leading to SM reduction restores RhoA membrane binding in ASMko synaptosomes (Supplementary figure C). These results complement those obtained in wt synaptosomes incubated with SM (Figure 3C). We also show that dexamethasone treatment, which rescues molecular and morphological alterations of ASMko dendritic spines *in vitro* and *in vivo* and improves functional deficits, acts through a mechanism that involves the transcriptional upregulation of the SM catabolic enzyme NSM (Figures 5C and 6A).

POINT 3. *-By a mechanistic point of view a possible involvement of lipid-rafts is SM-mediated alteration of dendrites spine physiology is only speculated in the discussion. Direct evidence in this direction should be provided.*

We now provide with the analysis of RhoA partitioning in rafts. We observe that the presence of RhoA diminishes in rafts from ASMko synaptosomes compared to wt (Supplementary figure F). Since rafts have been related with the stability of proteins we speculate that the alteration of RhoA

distribution in these membrane domains might contribute to its reduced levels and membrane binding. This is now discussed in page 14.

POINT 4. -*The authors used synthetic glucocorticoids in attempt to demonstrate a rescue of normal SM post-synaptic levels, Rho membrane binding and behavioural phenotype. The results are not convincing at all.*

There are no evidence that dexamethasone is specifically acting on SM levels in postsynaptic membranes of treated mice.

We showed that SM levels are reduced in ASMko synaptosomes treated with dexamethasone *in vitro* (Figure 5B) and in synaptosomes derived from ASMko mice orally treated with dexamethasone (Figure 6A). Further demonstrating the specific action of dexamethasone on SM metabolism we now report that dexamethasone treatment increases the mRNA and protein levels and activity of the SM catabolic enzyme, NSM, *in vitro* and *in vivo* (pages 10 and 12, Figures 5C and 6A).

The SM reduction in the brain of treated mice is very small, the increased membrane binding of Rho is even smaller.

As indicated in Figure 6B we observe a 36.7% SM reduction and 1.3-fold increased RhoA membrane attachment in synaptosomes of dexamethasone treated ASMko females. We do not think these differences are minor: they are statistically significant and are accompanied by normalization of dendritic spine size, enhanced neuronal survival and functional motor and memory improvement (Figures 6C and D and supplementary figure AB and AC).

The behavioral phenotype was monitored by only vertical pole test that showed a very limited improved coordination in only 63% of treated females.

Statistical analysis of the data with an appropriate test (chi-squared) indicates that the motor improvement in the treated females is significant with respect to the non treated animals. Instead, the difference is not any more significant between wt and treated ASMko females (Supplementary figure AC and detailed in this figure legend) indicating the reversion of the disease anomalies.

More then one behavioural test should be used.

To satisfy this reviewer´ query we have performed the Y- maze test, which assesses spatial memory governed by the hippocampus, in wt females and in ASMko females treated or not with dexamethasone. The test evidenced the poor performance of non treated ASMko females with respect to wt. This was significantly improved in dexamethasone treated females (page 12, Figure 6D). We thank this reviewer for this suggestion that extend the positive effects of dexamethasone treatment not only to motor but also to memory abilities.

Why males did not respond to the treatment is not sufficiently discussed.

The new experiments performed indicate that dexamethasone treatment induces a greater increase in NSM protein levels in synaptosomes derived from ASMko females than from males (compare Figure 6A with supplementary figure E). This might be the reason why, although there is a tendency to SM reduction in the dexamethasone treated ASMko males, this is not statistically significant. We report these data in page 11 and propose that the different level of expression of glucocorticoid receptors in females and males could be responsible for the different response (page 16).

Then, most of the references cited are very old (Okazaki et al., 1989; Ramachandran et al., 1990; Stumpf et al 1989), these references have shown reducing SM levels only in non neuronal cells and claim that synthetic glucocorticoids cross the BBB with very low efficiency.

Okazaki, Ramachandran and colleagues were the first reporting the effects of vitamin D3 or dexamethasone on NSM activity and SM levels in non neuronal cells. This is why we refer to their contribution. To our knowledge no other studies have been published on the effects of

dexamethasone in the levels of SM in neurons. We believe the contribution of our work in this regard is thus novel. On the other hand, we now include more updated references describing that dexamethasone, although with low efficiency, is able to cross the BBB (pages 11 and 15).

Many experiments lack of the necessary controls (loading controls in the coIPs experiments in fig 4.

These controls have been added (Figure 4C)

….treatment of wild-type both cells and mice with glucocorticoid drugs in figs 5 and 6).

We have treated wt synaptosomes, wt cultured neurons and wt mice with dexamethasone in the same conditions used for ASMko samples. We have not observed significant changes in SM levels, RhoA membrane binding or phalloidin levels in dendritic spines. These results are included as Supplementary figure D and mentioned in pages 10 and 11.

Minor points: -In many figures key information are missing. This results in a difficult reading of the data. For example:

Fig 1 B, which is the brain region analyzed?

We have now clarified in the panel and in the legend of Figure 1B that the CA1 stratum radiatum region belongs to the hippocampus.

Fig 2 A, in the quantification of lipids which synaptosomal fraction has been analyzed?

To quantify lipids in the postsynaptic membranes we performed an additional step in the synaptosomal purification protocol that is described in Methods (page 21). We confirmed that the final fraction obtained was enriched in postsynaptic markers such as PSD95 and devoided of presynaptic markers such as synaptophysin (see figure 2A). The lipid quantification shown in Figure 2A corresponds to the postsynaptic enriched fraction.

Fig 1C. The MAP2 staining cannot be distinguished. The images are of poor quality. Moreover, the small decrease in the fluorescent intensity (phalloidin) displayed in the graph is not representative of the reduction that can be detected by phalloidin staining in the fluorescence microscopy.

We now include, in all epifluorescence analysis for phalloidin staining, better quality images that also show the co-staining with the dendritic marker MAP2 and the postsynaptic marker PSD95 (new figures 1C, 2B and C, 5E and supplementary figure AC). Phalloidin associated fluorescence was quantitated in dendritic spines identified, by triple labelling immunofluorescence, as PSD95 positive protrusions derived from the dendritic shaft, which was labelled with MAP2. Mean intensity in spines was calculated per area unit. This is now indicated in methods (page 23).

Fig. 3B. Tubulin is not the best loading control for membrane fractions.

Although we agree with this reviewer that a membrane protein would be more appropriate as a loading control for membranes, little have been characterized on whether SM-induced alterations affect the levels of other synaptic membrane proteins in ASMko mice. This is why we decided to use a standard loading control such as tubulin, which levels are not altered in the absence of ASM. In addition, we always quantified protein amount in each sample by the BCA assay and monitored the Western blot transfer efficiency by staining of the nitrocellulose membranes with Red poinceau.

Fig. 6. Again the panels in the figure lack of details that make the figure hard to read

We have now included text panels in the figure to ease the reading.

Pag. 11. The authors claim that dexamethasone is currently prescribed for oral treatment of human diseases, key references lacking

We have now added some references about the use of dexamethasone in human diseases such as meningitis, vitiligo or Crohn´s disease (pages 11 and 16).

ANSWERS TO REVIEWER 3

We thank this reviewer for appreciating our experimental systems as excellent models to study neuronal dysfunction in Niemann Pick type A disease. We also thank him/her for considering that our analyses provide interesting new insights into the effects of sphingomyelin on dendritic spine morphology and may indicate a mechanism underlying neuronal dysfunction in disease. We have addressed his/her queries as follows:

POINT 1. *Pharmacologic activation of neutral sphingomyelinase is shown to rescue the dendritic phenotype of neurons in culture and improve functional deficits in ASM knockout mice. Since phenotypic rescue in vivo is accomplished by dexamethasone treatment, a manipulation that has many effects in the CNS, the authors should demonstrate that treatment reverses the dendritic spine defects shown in panels 1A,B.*

We have addressed the influence of dexamethasone treatment in dendritic spine defects by electron microscopy analysis of the hippocampus. The treatment restored dendritic spine size leading to a significant increase (36%) in the treated compared to non treated ASMko females (page 12, Figure 6C). Moreover, we now show that *in vivo* dexamethasone treatment not only reduces SM but also increases NSM protein and mRNA levels in synaptosomes (page 12, Figure 6A). These results confirm *in vivo* the ability of NSM activation to rescue the dendritic phenotype.

POINT 2. *The status of additional pathologies present in the brains of these animals at the end of treatment, such as neuron loss, should be commented upon.*

We have now monitored neuronal death in the cerebellum, since this is the only brain area where neuronal loss is evident at 4 months of age in ASMko mice (Macauley et al., 2008). Dexamethasone treatment enhanced Purkinje cell survival to a significant extent (59%) (page 12, supplementary figure AB).

A few other points should be addressed to strengthen the report.

What accounts for the decrease of mGluR levels in wt synaptosomes after treatment with SM (fig 4B)? Is there another fraction to which the receptors have translocated?

We have data indicating that high levels of SM destabilize mGluRs at the neuronal surface leading to increased internalization in ASMko neurons and wt neurons treated with SM. We propose that in the experiments in which we add SM to wt synaptosomes, mGluRs are internalized and degraded to a greater extent by lysosomes, which are present in our synaptosomal preparations. This could account for the reduced mGluR5 levels observed. We are currently studying this mechanism and its functional consequences in detail to complete another manuscript. Therefore, we kindly ask this reviewer to let us keep these data unpublished for the moment. We nonetheless discuss this issue in the present manuscript (page 14).

In fig 6B, it's curious that DM treatment seems to have its greatest effect on RhoA levels in the supernatant fraction rather than the pellet. Why is this? Is there a significant difference when one analyzes RhoA only in the pelleted fraction after DM treatment?

There is also a significant difference when analysing RhoA levels in the pelleted fractions (2.1-fold more in dexamethasone treated). We provide with more examples of the amount of RhoA in the

© EMBO

pellet and the quantification (n=7) in the figure below for this reviewer assessment. However, we believe that comparing supernatant and pelleted fractions in the same samples (as we have done in all RhoA binding experiments) is a more accurate way of detecting differences in membrane binding since it would not be affected by potential loading variations. This is why we would prefer to leave these data in the final figure 6B.

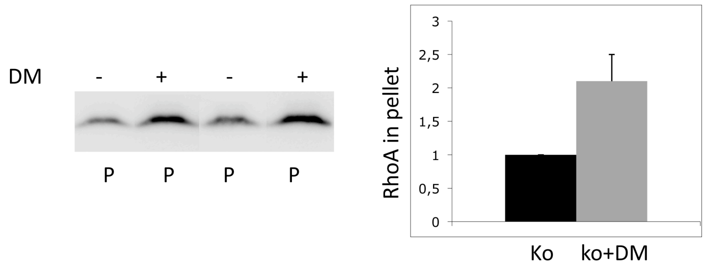

Loading controls are missing from a couple of western blots (figures 2a, 3Ba).

Loading controls with tubulin have been added in Figure 3Ba. Figure 2a shows fractions derived from the same sample submitted to a purification protocol in which we enriched in postsynaptic densities. Therefore, it is difficult to find common markers that can be used as loading controls. Nonetheless, we normalized by total amount of protein monitored by the BCA assay in each fraction.

In Fig 4, panel 4Cc is referred to as panel 4D in the legend.

Corrected now in figure 5Cc

3rd Editorial Decision 31 October 2013

Thank you for the submission of your revised manuscript to EMBO Molecular Medicine. We have now received the enclosed reports from the referees that were asked to re-assess it. As you will see the reviewers are now globally supportive and I am pleased to inform you that we will be able to accept your manuscript pending the following few remaining issues noted by Reviewer 2 that I would ask you to deal with appropriately.

Reviewer 2 would like to you to discuss why ASM loss has different impacts on different neuron populations. Also, s/he notes that flotillin (or other suitable cholesterol-rich domain marker) should be assayed to determine the amount of raft-like membranes in the synaptosomal membrane fraction. I agree that, although experimental support would be ideal, you should at least clarify the apparenbt dicrepancy the Reviewer refers to. Finally, the Reviewer would also like you to add new images of cerebellum showing similar regions in Ko and Ko + DM .

Please submit your revised manuscript within two weeks. I look forward to seeing a revised form of your manuscript as soon as possible.

***** Reviewer's comments *****

Referee #1 (Remarks):

Response to critique adequate.

Referee #2 (Remarks):

The manuscript is now improved and satisfies most of our criticism. However, some issues are still not clear or not sufficiently addressed:

- Fig. 1 . The authors claim "the absence of ASM has a broad impact in dendritic spines of different neuronal populations ranging from spine loss in the cortex to reduced size in the hippocampus and cerebellum."
Since this appears to be a general mechanism the authors should speculate in discussion section why ASM loss has a different impact on different population of neurons.

-Supporting information Fig 6. What about rafts amount in synaptosomal membrane fraction? The authors should blot flotillin to quantify the variation in raft abundance. Since SM positively regulates raft formation I would expect an increase in raft domains in ASMKo and as consequence an increase in Rho membrane binding to raft domains in ASMKO synaptosomes. Instead, the authors found a reduction in Rho membrane binding associated to a reduction in Rho raft binding in ASMKo synaptosomes.
If authors want to conclude that rafts are involved in Rho membrane binding loss they should clarify this discrepancy.

-Supporting information 1B. Add new pictures of cerebellum showing similar regions in Ko and Ko + DM

Referee #3 (Comments on Novelty/Model System):

This is a nice paper that provides a mechanistic explanation of the occurrence of dendritic spine defects in ASM deficient mice, and then leverages this mechanistic understanding to test a new therapeutic strategy. The additional, new data incorporated into the revision significantly strengthens the report.

Referee #3 (Remarks):

The authors have addressed all of my concerns from the prior submission.

2nd Revision - authors' response 20 November 2013

Referee 2

- Fig. 1 . The authors claim "the absence of ASM has a broad impact in dendritic spines of different neuronal populations ranging from spine loss in the cortex to reduced size in the hippocampus and cerebellum." Since this appears to be a general mechanism the authors should speculate in discussion section why ASM loss has a different impact on different population of neurons.

Our data reflect a similar effect of ASM loss in dendritic spines that is the reduction in their size. What varies between neuronal populations is the extent of size reduction, which in neurons of the cortex is such that leads to the disappearance of these membrane protrusions. We do not have a real explanation for this observation (to our knowledge there are no studies reporting a differential expression of ASM in distinct neuronal populations or different lipid compositions in their dendrites). Nevertheless, we can speculate that it might be due to the different time of exposure or sensibility (i.e. basal levels of SM, robustness of compensatory mechanisms) to the increased SM levels. We now comment this issue in the discussion section (underlined text page 14).

© EMBO

-Supporting information Fig 6. What about rafts amount in synaptosomal membrane fraction? The authors should blot flotillin to quantify the variation in raft abundance. Since SM positively regulates raft formation I would expect an increase in raft domains in ASMKo and as consequence an increase in Rho membrane binding to raft domains in ASMKO synaptosomes. Instead, the authors found a reduction in Rho membrane binding associated to a reduction in Rho raft binding in ASMKo synaptosomes. If authors want to conclude that rafts are involved in Rho membrane binding loss they should clarify this discrepancy.

In the new Supporting information 6 we include the Western blot analysis of Flotillin in the sucrose gradient fractions after detergent extraction of synaptosomes derived from wt or ASMko mice. In contrast to RhoA the presence of Flotillin in the floating fractions corresponding to rafts is not affected by ASM deficiency. This rules out that more SM increases raft abundance in the ASMko synapses. More SM would nevertheless change the lipid balance in these microdomains affecting the affinity of certain proteins like RhoA but not of others. This is now discussed in the underlined text on page 15.

-Supporting information 1B. Add new pictures of cerebellum showing similar regions in Ko and Ko + DM.

We have now done so in the new figure 1B of supporting information.
