# Supplementary material for: Pharmacological reversion of sphingomyelin-induced dendritic spine anomalies in a Niemann Pick disease type A mouse model
Source: EMBO Mol Med. 2014 Jan 21;6(3):398–413. doi: 10.1002/emmm.201302649 (PMC3958313; doi:10.1002/emmm.201302649)
Supplement: Supplementary file 2 [file emmm0006-0398-sd2.pdf]

SUPPORTING INFORMATION FIGURE 1

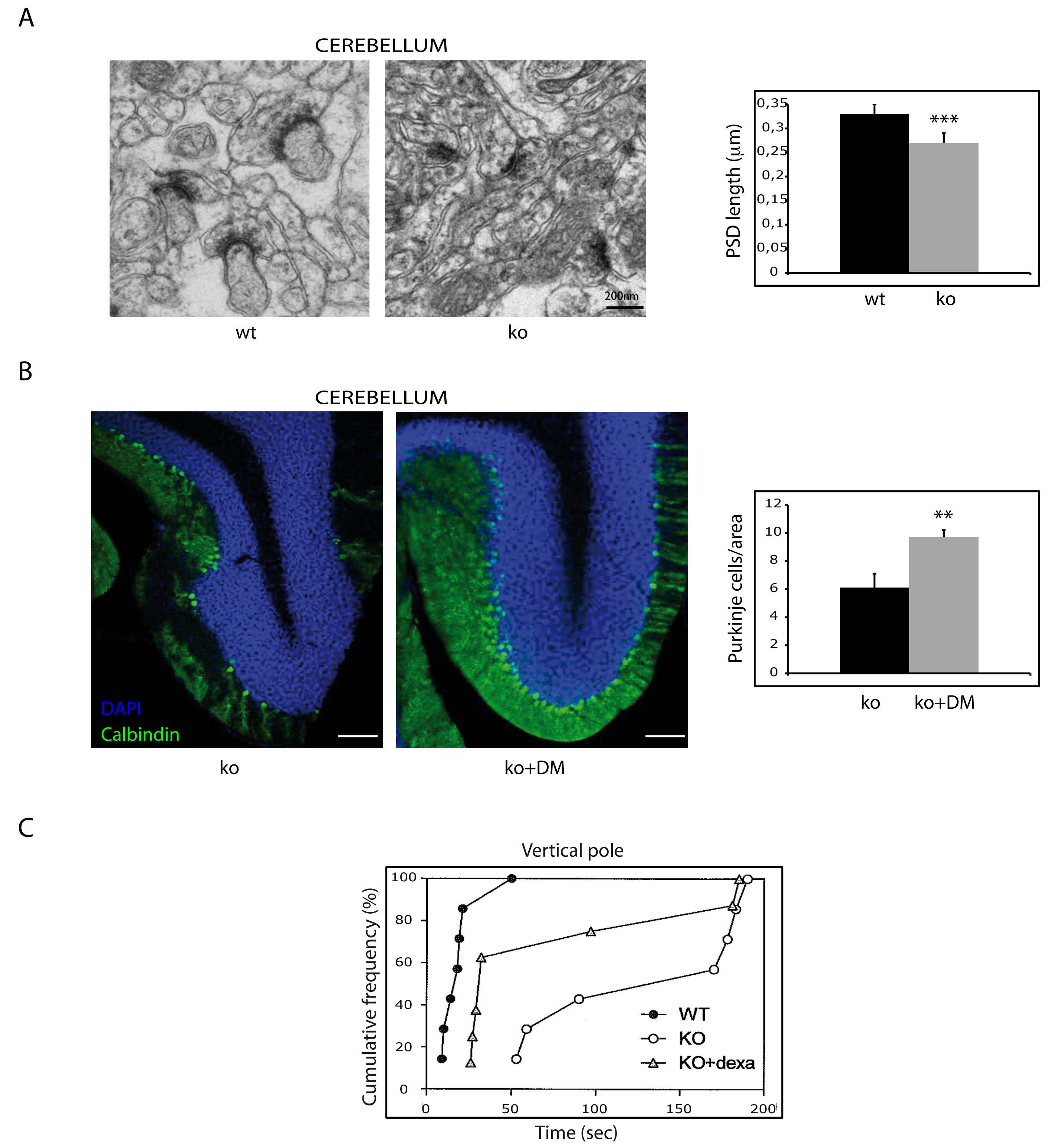

Supporting information Figure 1

Alterations in the cerebellum of ASMko mice and reversion by dexamethasone treatment.

A. Electron micrographs of synapses in the molecular layer of the cerebellum from wt and ASMko mice. Graph shows mean ± SD of PSD length in μm in wt and ASMko mice (n= 70 synapses in each of 3 mice per genotype) (p=0.0001). B. Purkinje cells identified by Calbindin labelling (in green) in the cerebellum of ASMko females treated or not with dexamethasone. Dapi in blue. Bars: 100 μm. Graph shows number of calbindin positive neurons per area unit (n=3, p=0.006). C. Results of the vertical pole test in wt, ASMko and dexamethasone ASMko treated females. Graph shows the cumulative frequency with respect to the total time (in seconds) employed by the mice to descend the pole. Statistical analysis with the chi-squared test confirmed that the differences were significant between wt and ASMko non-treated females (p=0.0009) and between ASMko treated and non-treated females (p=0.0376). However, the difference was not significant between ASMko-treated and wt females (p=0.193) (n=7).
