# Supplementary material for: Pharmacological reversion of sphingomyelin-induced dendritic spine anomalies in a Niemann Pick disease type A mouse model
Source: EMBO Mol Med. 2014 Jan 21;6(3):398–413. doi: 10.1002/emmm.201302649 (PMC3958313; doi:10.1002/emmm.201302649)
Supplement: Supplementary file 3 [file emmm0006-0398-sd3.pdf]

**SUPPORTING INFORMATION FIGURE 2**

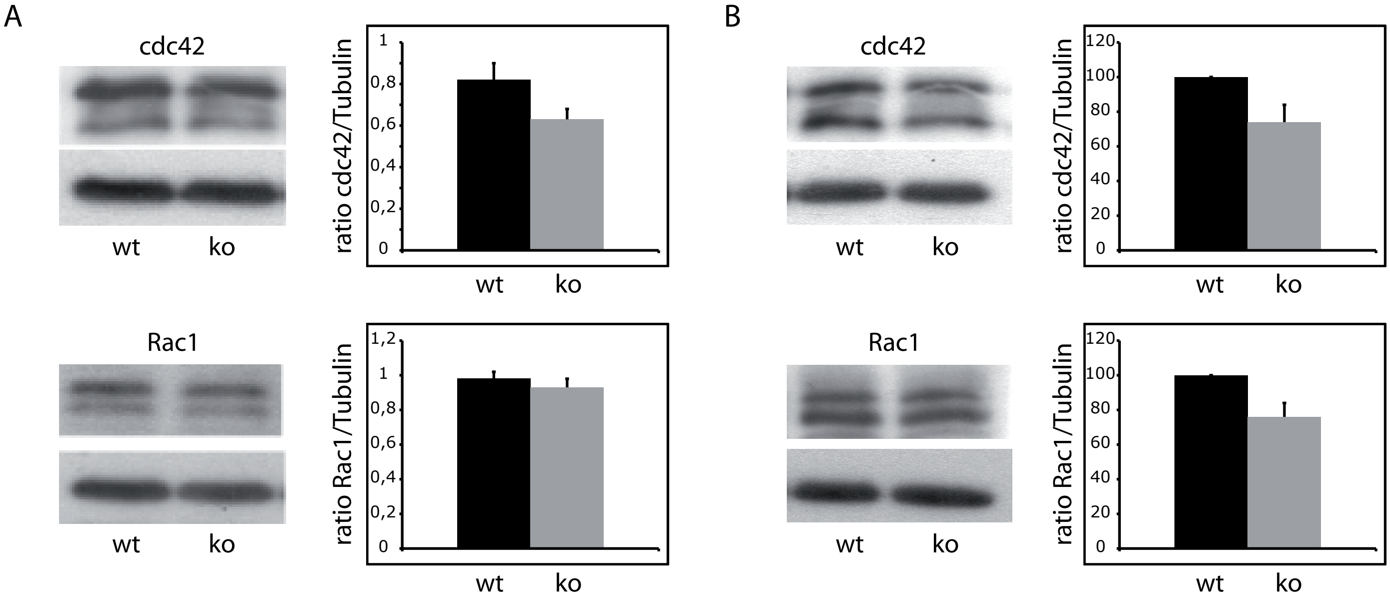


**Supporting Information Figure 2**
**Levels and membrane attachment of cdc42 and Rac1 are not altered in ASMko synaptosomes.**
Western blot of cdc42, Rac1 and tubulin levels in total (A) and membrane extracts (B) from wt and ASMko synaptosomes. Graphs show mean ± SD of cdc42 and Rac1 levels in ASMko and wt conditions normalized to tubulin in arbitrary units. For membrane extracts ASMko values were referred to those wt that were considered as 100 (n=3).
