# Supplementary material for: Pharmacological reversion of sphingomyelin-induced dendritic spine anomalies in a Niemann Pick disease type A mouse model
Source: EMBO Mol Med. 2014 Jan 21;6(3):398–413. doi: 10.1002/emmm.201302649 (PMC3958313; doi:10.1002/emmm.201302649)
Supplement: Supplementary file 4 [file emmm0006-0398-sd4.pdf]

SUPPORTING INFORMATION FIGURE 3

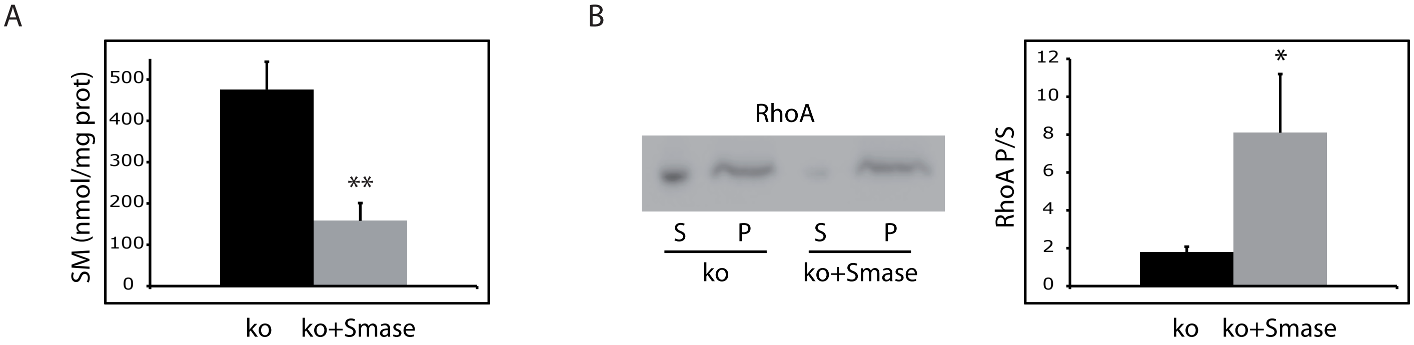

Supporting Information Figure 3.
Sphingomyelinase treatment restores RhoA membrane binding in ASMko synaptosomes.
A. Mean ± SD of SM levels (nmol/mg protein) in ASMko synaptosomes treated or not with sphingomyelinase (n=3, p=0.016). B. Western blots of RhoA in supernatants (S) and pellets (P) after 100000g centrifugation of ASMko synaptosomes treated or not with sphingomyelinase. Graph shows mean ± SD of RhoA ratio pellet/supernatant (n=3; p= 0.032).
