# Supplementary material for: Pharmacological reversion of sphingomyelin-induced dendritic spine anomalies in a Niemann Pick disease type A mouse model
Source: EMBO Mol Med. 2014 Jan 21;6(3):398–413. doi: 10.1002/emmm.201302649 (PMC3958313; doi:10.1002/emmm.201302649)
Supplement: Supplementary file 5 [file emmm0006-0398-sd5.pdf]

SUPPORTING INFORMATION FIGURE 4

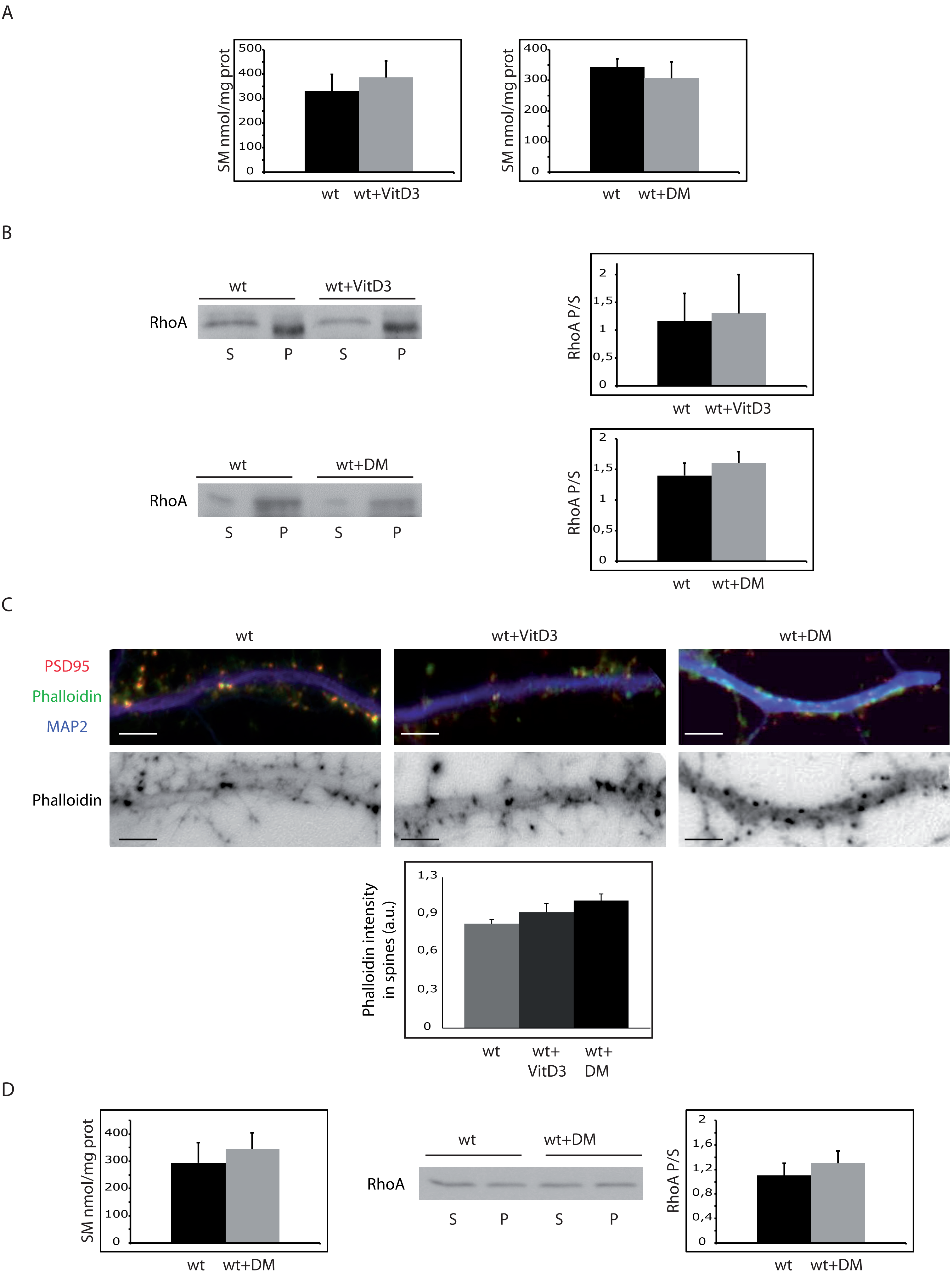

Supporting Information Figure 4.
α, 25-dihydroxivitamin D3 or dexamethasone treatments have no significant effects in wt synaptosomes, wt cultured neurons or wt mice.
A. Mean ± SD of SM levels (nmol/mg protein) in wt synaptosomes treated or not with 1α, 25-dihydroxivitamin D3 (VitD3) or dexamethasone (DM) (n=3).
B. Western blots showing RhoA levels in supernatants (S) and pellets (P) after 100000g centrifugation of wt synaptosomes treated or not with VitD3 or DM. Graphs show mean ± SD of RhoA ratio pellet/supernatant (n=3).
C. Top: Dendrites from wt neurons non treated or treated with VitD3 or DM stained for MAP2 (blue), PSD95 (red) and phalloidin (green); bottom: phalloidin staining only. Graph shows mean ± SD of phalloidin fluorescence intensity per spine area (n= 250 dendritic spines from 3 independent cultures). Bars: 5 μm.
D. Graph in the left shows SM levels (in nmol/mg protein) in synaptosomes from wt females treated or not with dexamethasone (n=10). Central panel shows Western blots of RhoA in supernatants (S) and pellets (P) after 100000g centrifugation of synaptosomes from wt females treated or not with dexamethasone. Graph at the right shows mean ± SD of the RhoA ratio pellet/supernatant (n=10).
