# Supplementary material for: Pharmacological reversion of sphingomyelin-induced dendritic spine anomalies in a Niemann Pick disease type A mouse model
Source: EMBO Mol Med. 2014 Jan 21;6(3):398–413. doi: 10.1002/emmm.201302649 (PMC3958313; doi:10.1002/emmm.201302649)
Supplement: Supplementary file 6 [file emmm0006-0398-sd6.pdf]

SUPPORTING INFORMATION FIGURE 5

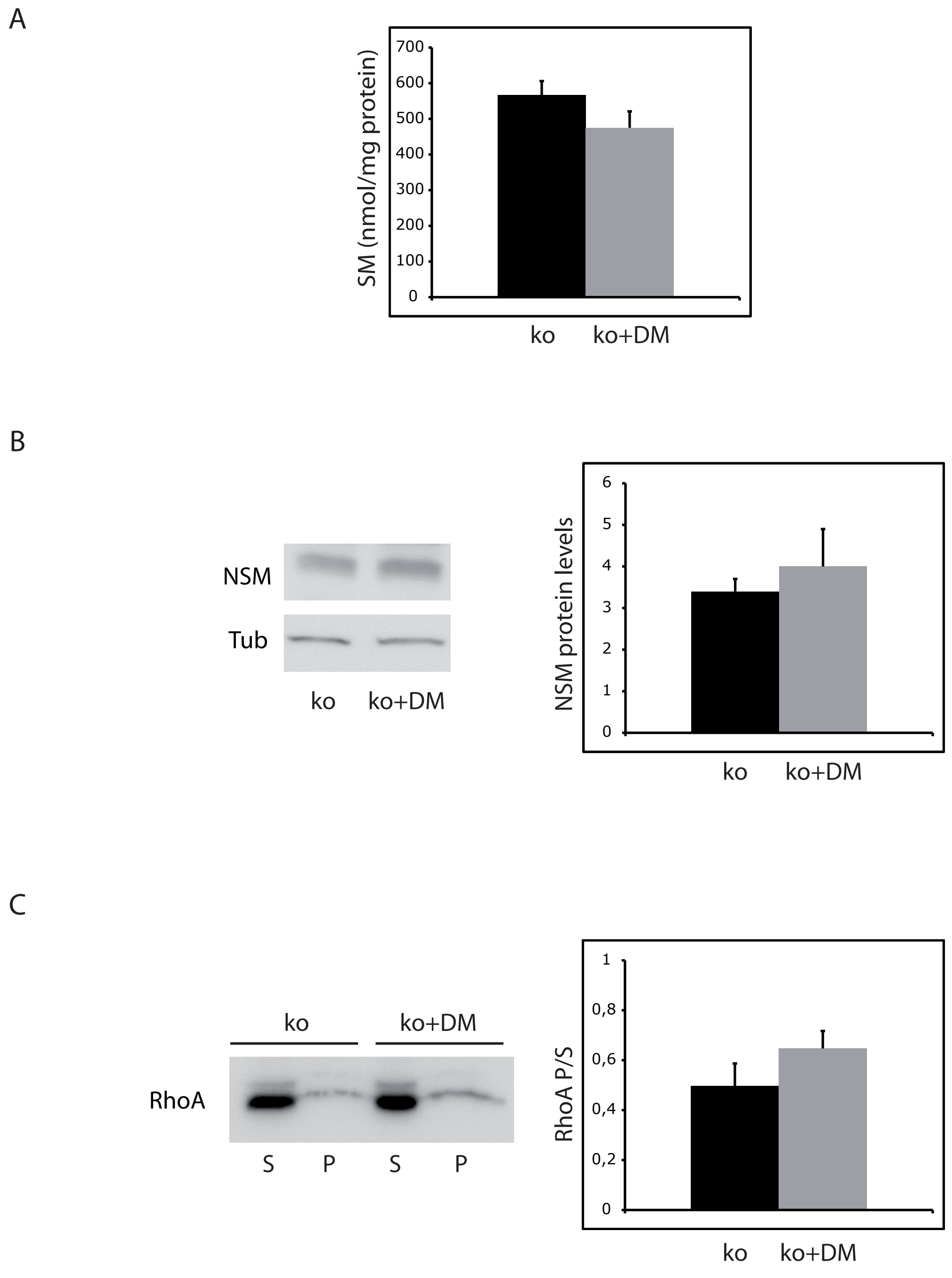

Supporting Information Figure 5

Dexamethasone treatment of ASMko males does not alter SM levels, NSM expression or RhoA membrane binding in synaptosomes.

A. Mean ± SD of SM levels (nmol/mg protein) in synaptosomes from ASMko males treated or not with dexamethasone (n=10). B. Western blot of NSM and tubulin levels in synaptosomes derived from ASMko males treated or not with dexamethasone. Graph shows mean ± SD of NSM levels normalized to tubulin (n=10). C. Western blot of RhoA in supernatants (S) and pellets (P) after 100000g centrifugation of synaptosomes from ASMko males treated or not with dexamethasone. Graph shows mean ± SD of the RhoA ratio pellet/supernatant (n=10).
