# Supplementary material for: Pharmacological reversion of sphingomyelin-induced dendritic spine anomalies in a Niemann Pick disease type A mouse model
Source: EMBO Mol Med. 2014 Jan 21;6(3):398–413. doi: 10.1002/emmm.201302649 (PMC3958313; doi:10.1002/emmm.201302649)
Supplement: Supplementary file 7 [file emmm0006-0398-sd7.pdf]

SUPPORTING INFORMATION FIGURE 6

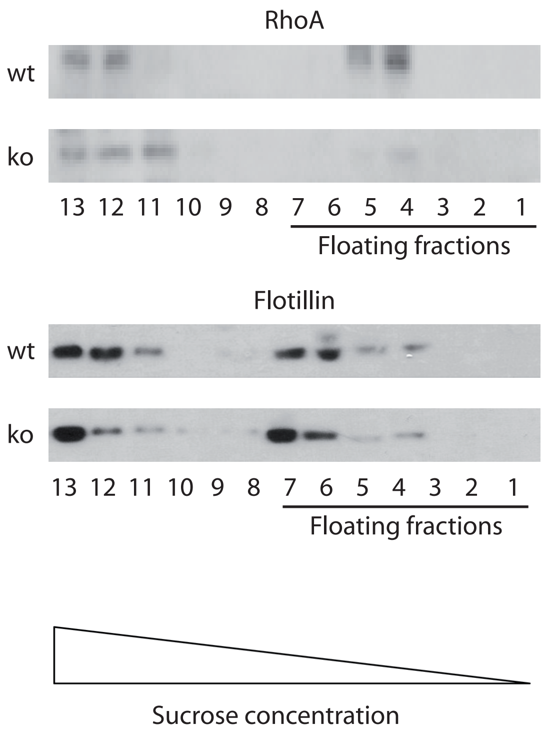

Supporting Information Figure 6.
RhoA distribution in rafts is altered in ASMko synaptosomes. RhoA and Flotillin membrane partitioning analyzed by Western blot of the fractions from the sucrose gradients after cold TritonX-114 extraction of synaptosomes from wt or ASMko mice. Light (floating) fractions 1-7 correspond to rafts.
