# Supplementary material for: Pharmacological reversion of sphingomyelin-induced dendritic spine anomalies in a Niemann Pick disease type A mouse model
Source: EMBO Mol Med. 2014 Jan 21;6(3):398–413. doi: 10.1002/emmm.201302649 (PMC3958313; doi:10.1002/emmm.201302649)
Supplement: Supplementary file 8 [file emmm0006-0398-sd8.pdf]

SUPPORTING INFORMATION FIGURE 7

A

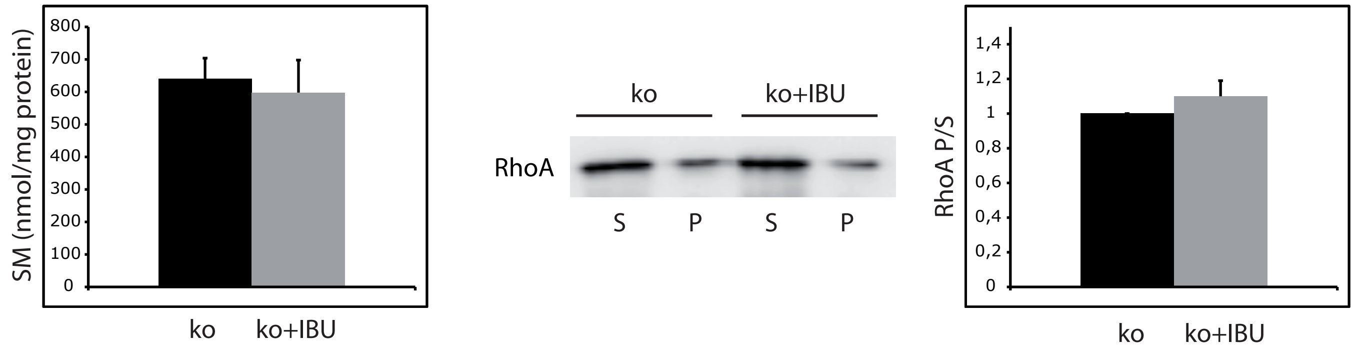

B

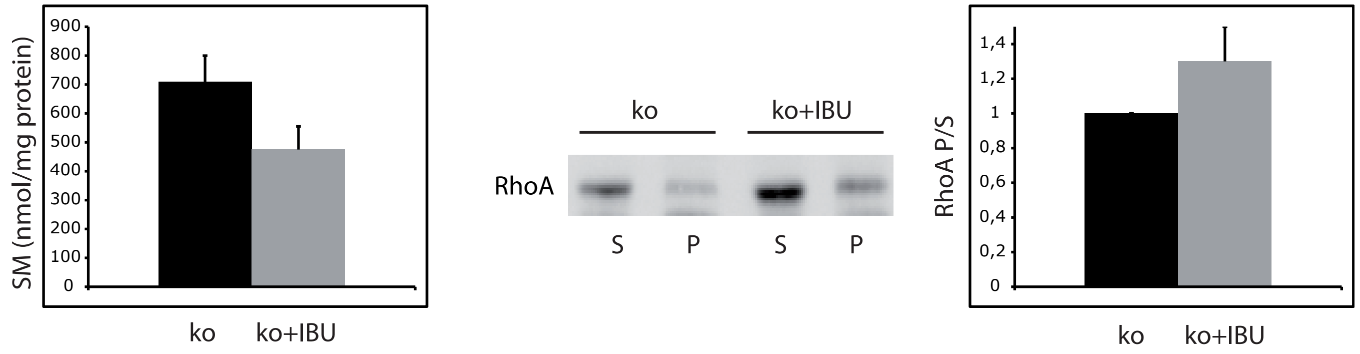

Supporting Information Figure 7.

In vitro and in vivo treatment with ibuprofen does not significantly alter SM levels or RhoA membrane binding in ASMko synaptosomes.

A. Mean ± SD of SM levels (nmol/mg protein) in ASMko synaptosomes treated or not with ibuprofen (n=3). Central panel shows Western blots of RhoA in supernatants (S) and pellets (P) after 100000g centrifugation of synaptosomes from ASMko synaptosomes treated or not with ibuprofen. Graph at the right shows mean ± SD of the RhoA ratio pellet/supernatant in ASMko synaptosomes treated with ibuprofen referred to non treated that were considered as 1 (n=3). B. Mean ± SD of SM levels (nmol/mg protein) in synaptosomes from ASMko mice orally treated or not with ibuprofen (n=10). Western blots of RhoA in supernatants (S) and pellets (P) after 100000g centrifugation of synaptosomes from ASMko mice orally treated or not with ibuprofen. Graph shows mean ± SD of the RhoA ratio pellet/supernatant in synaptosomes derived from ASMko females treated with ibuprofen referred to non treated that were considered as 1 (n=7).
